# Verwey transition as evolution from electronic nematicity to trimerons via electron-phonon coupling

**DOI:** 10.1126/sciadv.adf8220

**Published:** 2023-06-09

**Authors:** Wei Wang, Jun Li, Zhixiu Liang, Lijun Wu, Pedro M. Lozano, Alexander C. Komarek, Xiaozhe Shen, Alex H. Reid, Xijie Wang, Qiang Li, Weiguo Yin, Kai Sun, Ian K. Robinson, Yimei Zhu, Mark P.M. Dean, Jing Tao

**Affiliations:** ^1^ Condensed Matter Physics and Materials Science Division, Brookhaven National Laboratory, Upton, NY 11973, USA.; ^2^ Department of Physics and Astronomy, Stony Brook University, Stony Brook, NY 11794-3800, USA.; ^3^ Max Planck Institute for Chemical Physics of Solids, Nöthnitzer Street 40, 01187 Dresden, Germany.; ^4^ SLAC National Accelerator Laboratory, Menlo Park, CA 94025, USA.; ^5^ Department of Physics, University of Michigan, Ann Arbor, MI 48109, USA.; ^6^ London Centre for Nanotechnology, University College, London WC1E 6BT, UK.

## Abstract

Understanding the driving mechanisms behind metal-insulator transitions (MITs) is a critical step toward controlling material’s properties. Since the proposal of charge order–induced MIT in magnetite Fe_3_O_4_ in 1939 by Verwey, the nature of the charge order and its role in the transition have remained elusive. Recently, a trimeron order was found in the low-temperature structure of Fe_3_O_4_; however, the expected transition entropy change in forming trimeron is greater than the observed value, which arises a reexamination of the ground state in the high-temperature phase. Here, we use electron diffraction to unveil that a nematic charge order on particular Fe sites emerges in the high-temperature structure of bulk Fe_3_O_4_ and that, upon cooling, a competitive intertwining of charge and lattice orders arouses the Verwey transition. Our findings discover an unconventional type of electronic nematicity in correlated materials and offer innovative insights into the transition mechanism in Fe_3_O_4_ via the electron-phonon coupling.

## Teaser

Verwey transition could be evoked by the emergence of a nematic order interacting with specific phonon modes upon cooling.

## INTRODUCTION

The Verwey transition in magnetite, Fe_3_O_4_, signaled by a sudden decrease in electrical conductivity by a factor of 100 upon cooling, is known to be associated with a structural phase transition from cubic to monoclinic at a temperature around the Verwey temperature, *T*_v_ = 125 K (*1*–*3*). As the first transition metal oxide material with proposed charge order–induced MIT and structural transitions, searching for the origin of charge ordering and charge-lattice interactions in Fe_3_O_4_ have captivated tremendous research efforts for nearly a century (*4*–*6*). However, the role of charge ordering in this material has been a longstanding debate. On one hand, long-range charge order was argued to be a result, rather than a cause, at the critical point (*T*_v_) of the transition (*7*–*9*), leaving the mechanism for the phenomenon unresolved. On the other hand, short-range lattice distortion and electronic instability have been reported in the electron, x-ray, and neutron scattering studies (*10*–*14*), no experiments to date observe any long-range charge order above the critical temperature *T*_v_. Theoretical studies have even suggested that a potential long-range order would be unstable (*15*). Hence, the Verwey transition mechanism is a heavily clouded subject. The technical difficulty of searching charge order at and above *T*_v_ may lie in the material’s integrity including oxygen stoichiometry (*16*, *17*), distinct surface, and bulk quality and properties (*18*–*20*).

In this work, we study the symmetry breaking on electronic structure and explore the interaction between electrons and lattice in Fe_3_O_4_ using electron scattering. The extra reflections observed in the diffraction pattern indicate a long-range charge order, i.e., nematic phase, embedded in the high-temperature phase. Using femtosecond laser pulses, we observed a strong electron-phonon coupling in the transient state with megaelectron volt (MeV) electron probe. We provide a linkage between the nematic order and trimeron order to advance the understanding of Verwey transition.

## RESULTS

### Electron diffraction results using a TEM

**Fig. 1.**
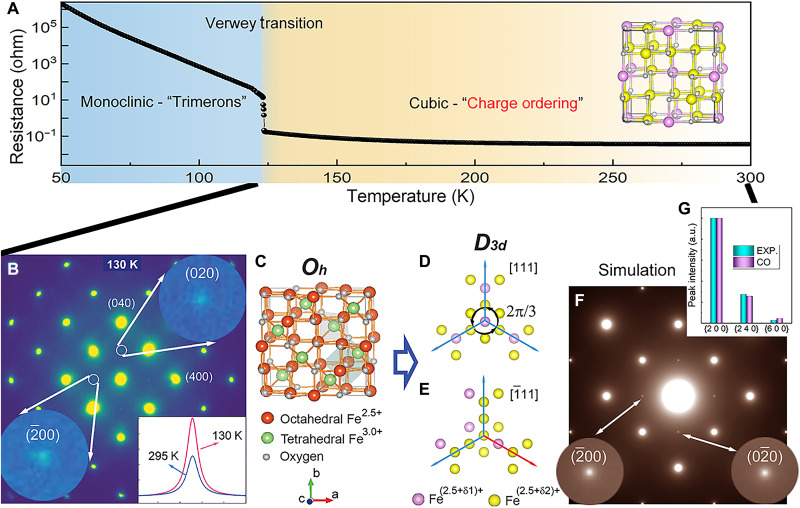
A discovery of charge ordering in Fe_3_O_4_ crystals at the temperatures above the Verwey temperature. (**A**) A resistance measurement from single-crystal Fe_3_O_4_ shows a sharp transition at *T*_v_ ~ 123 K, measured from sample 2. Inset is a charge ordering model in the cubic phase. (**B**) Upon cooling, {200} reflections, which are forbidden in the cubic structure, can be seen in the electron diffraction pattern, taken from sample 1. The inset shows the intensity profiles of the forbidden {200} reflections at 130 and 295 K. (**C**) A crystal model of the cubic Fe_3_O_4_, *O_h_* is the point group for the cubic structure. (**D** and **E**) Charge distribution on the octahedral Fe sites with different valence charge values, i.e., Fe^(2.5+δ1)+^ and Fe^(2.5+δ2)+^ along [111] and $\left[\bar{1}11\right]$ direction. The threefold rotational symmetry is preserved in [111] direction shown in (D), and it is broken in (E). *D_3d_* is the corresponding point group considering rotational symmetry breaking in the charge ordering model. (**F**) Simulation of electron diffraction patterns based on the charge ordering model are shown in (D) and (E). (**G**) Normalized intensities of experimentally measured from {200}, {240}, and {600} reflections are compared to the results of simulations based on the charge ordering model using dynamic electron diffraction methods, showing a consistency. EXP. is the experimental data, and charge ordered (CO) is the calculation result on the basis of CO model. a.u., arbitrary unit.

The transmission electron microscope (TEM) experiment was performed on two single crystals with slightly different stoichiometries (sample 1: Fe_3(1+0.0005)_O_4_ and sample 2 Fe_3(1–0.001)_O_4_; see electronic resistance measurement in fig. S1 and the Supplementary Materials for additional details). The diffraction patterns as a function of temperature from samples 1 and 2 are presented in figs. S2 and S3. Both samples present an abrupt increase in the electronic resistance measurement at *T*_v_. At low temperatures, the superlattice (SL) reflections corresponding to the superstructure with trimerons are observed in both samples in the diffraction patterns. The slightly different transition temperatures are due to the different stoichiometries of the samples, and all these features indicate that samples 1 and 2 are high-quality crystals and qualitatively the same, as shown in Fig. 1B. For simplicity, all the reflections and orientations here will be defined with reference to the cubic structure. It is very unexpected that the {200} reflections with weak intensity in the <001> zone electron diffraction pattern, which should be forbidden for the 16*d* site of Fe ions in the cubic structure (space group: $Fd\bar{3}m$, no.227) (*21*, *22*), are present over a wide range of temperatures above *T*_v_. The “forbidden” reflections are sharp, with similar widths to the main Bragg peaks, i.e., a long-range order associated with the forbidden reflections is likely present. We have examined many sample flakes with various sample thicknesses in the TEM, and we find that the {200} reflections are undetectable in the very thin areas but statistically dominant in the relatively thick areas (typically tens of nanometers in thickness). This indicates that the forbidden reflections are not from the sample surface due to the surface contamination or reconstruction but are intrinsic to the bulk. We also note that these forbidden reflections at [001] zone axis cannot be introduced by multiple scattering because the lack of a combination of any crystallographically allowed fundamental Bragg reflections to generate {200} reflections in the reciprocal space. To verify this point, the dynamic diffraction simulation was performed by considering the appropriate sample thickness and multiple scattering effects, and the corresponding simulation result is shown in fig. S4.

### The origin of the forbidden reflections

**Fig. 2.**
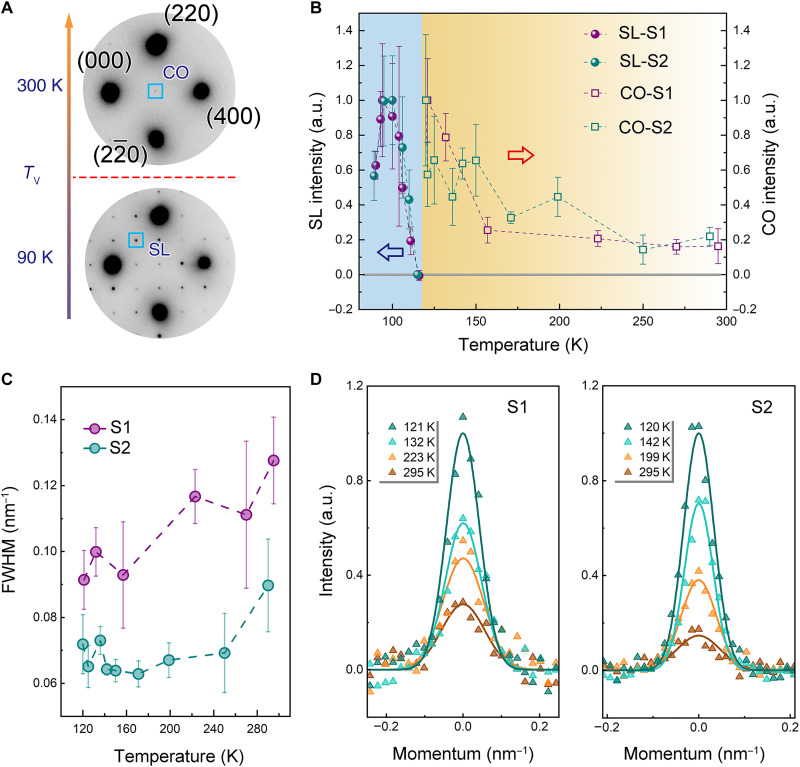
Evolution of charge order and lattice order measured by reflection intensities in electron diffraction patterns upon cooling in Fe_3_O_4_. (**A**) Electron diffraction patterns above and below Verwey temperature *T*_v_, with the appearance of the CO reflection in electronic nematic phase and SL reflection arising from the monoclinic structure with the trimeron charge order, respectively. (**B**) Intensity variations of CO and SL reflections are plotted as a function of temperature. Results were plotted from diffraction data obtained from two similar samples, sample 1 (S1) and sample 2 (S2). Error bars represent the SD in the mean of intensities from the equivalent reflections. (**C**) The variation of peak width, i.e., full width at half maximum (FWHM), of CO reflections measured from two samples upon cooling. The error bars of peak width plots were estimated on the basis of measurement uncertainties and the instrument broadening due to diffraction limits. (**D**) Line profile of CO reflection at different temperatures. The experiment data are shown as triangle symbols, and the solid lines are the fitted results.

First, because more reflections, e.g., {100} and {110}, would present in the case (see Fig. 2A) of the monoclinic phase (space group: *Cc*, no. 9), which occurs below *T*_v_, the appearance of only {200} reflections in Fig. 1B rules out the existence of a lower symmetry with the monoclinic structure embedded in the high-temperature phase. Second, we found that the {200} reflections could present when we assign different valence charges at octahedral Fe sites, which are widely considered as the candidate ions that induce the potential charge ordering (*3*). According to the $Fd\bar{3}m$ symmetry, we can preserve the threefold rotational symmetry along one of <111> families of directions as shown in Fig. 1D, while breaking the other three threefold rotational symmetries by assigning different valence charge at 16*d* Fe ions, as shown in Fig. 1E and fig. S5. This type of charge ordering converts the $Fd\bar{3}m$ space group to $R\bar{3}m$ space group and gives rise to {200} reflections in the diffraction simulations in Fig. 1F. According to the charge arrangement, there are four twin variants in the charge ordering models (see the Supplementary Materials for additional details and fig. S5). Dynamic electron diffraction simulations based on models with adjustable valence charge values were carried out and compared with experiments (Fig. 1G and fig. S6), revealing the charge differentiation between the two ordered Fe ions could be 0.05 e^−^.

We note that atomic displacements of octahedral Fe atoms with the same symmetry breaking indicated in Fig. 1, D and E, could also give rise to the forbidden reflections in diffraction patterns. Our dynamic electron diffraction simulations and the comparison with the experimental data reveal possible existence of a pure atomic displacement wave with an amplitude of ~0.05 Å (for the dynamic electron diffraction calculation on atomic displacement, see the Supplementary Materials for additional details and fig. S7). However, in that case, neutron diffraction would be sufficient to detect it with much better accuracy in ascertaining atomic positions and lattice symmetry by neutron diffraction techniques. Fe_3_O_4_ crystals are known to have diffuse scatterings in proximity to the Bragg peaks at the temperature just above *T*_v_ and the intensities of those diffuse scatterings are comparable to the forbidden {200} reflections in electron diffraction (the intensity comparison of diffuse scattering and {200} peaks are shown in fig. S8). Neutron diffraction results have reported the observations of the diffuse scattering but did not detect the {200} reflections at temperatures above *T*_v_ (*7*, *10*, *11*). These facts strongly suggest that, although we cannot rule out the possibility of lattice symmetry breaking, the intensity of the forbidden {200} reflections must mainly come from an electronic ordering. To quantitatively separate the electron and lattice contribution to {200} reflections, more experiments are desirable. Applying external anisotropic strain to tune the lattice structure can be insightful to explore the competition between electronic and lattice structures (*23*, *24*). In addition, the intrinsic electronic response to the lattice distortion can also be extracted via measuring the elastic shear modulus as shown in (*25*). Moreover, the inelastic light scattering is a beneficial tool to identify the lattice structure and electronic nature simultaneously across the Verwey transition (*26*).

In a prior resonant x-ray diffraction study, the observation of {200} reflections was ascribed to anomalous tensor scattering from the anisotropy of the local electron density on the 16*d* Fe ions (*27*). This occurs because the orbital orientation at the 16*d* site follows the $\bar{3}m$ crystallographic point group symmetry at these sites. For the electron scattering cross section, {200} reflections are extinct since the $Fd\bar{3}m$ space group symmetry is preserved. [the reflection conditions for $Fd\bar{3}m$ space group symmetry, *h *0 0: *h* = 4*n*; 0 *k *0: *k* = 4*n*; 0 0 *l*: *l* = 4*n*, *n* = integers (*28*)]. Thus, the anomalous scattering case discussed in (*27*) cannot explain what we observed in the electron diffraction data. In our charge ordering model as shown in Fig. 1 (D and E), the contribution of 16*d* Fe ions to {200} reflections are no longer canceled out due to the different charges among 16*d* Fe ions, which is the best solution in our study. Moreover, electron scattering is more sensitive to valence charge ordering than x-ray techniques due to its strong scattering power and the nature of interaction with electrostatic potentials in the sample (*29*). To examine the different sensitivities to Fe ions, we calculated the electron and x-ray form factors for Fe^2+^ and Fe^3+^ ions, as shown in fig. S9. The calculated result illustrates that, comparing with x-ray scattering, electron scattering is more sensitive to the disproportion of the valence electrons at low scattering angles, e.g., {200} reflections, which may explain why those forbidden reflections can be observed using electron diffraction. Furthermore, although the resonant x-ray scattering enhances the sensitivity to the electronic states (*30*), the small charge disproportion, i.e., ~0.05 e^−^, may be too weak to be detected using the resonant x-ray anisotropic tensor susceptibility mechanism in Fe_3_O_4_, as suggested in (*31*, *32*). Therefore, we conclude that the different sensitivities of the x-rays and electrons to the different types of electronic structures lead to a comprehensive study on electronic state in Fe_3_O_4_, i.e., anisotropic electron density state with different probabilities on 16*d* Fe ions.

### Electronic nematic phase above *T*_v_

Since the amount of the long-range–ordered lattice distortions here appears to be below the sensitivity of standard neutron diffraction experiments and the symmetry of the electron distribution is unambiguously broken from the cubic phase, the anisotropic electronic structure in Fe_3_O_4_ presents a paradigm of an electronic liquid crystal (ELC) phase at the temperature above *T*_v_. The ELC phases are quantum states of matter where strong interactions between electrons drive an instability that spontaneously breaks certain point group and space group symmetry of a crystal (*33*), i.e., describing a state with a modulated electronic structure. Among various ELC phases, we found that the most relevant one for our study is the electronic nematic phase, where certain rotational symmetry is spontaneously broken, while the translation and space inversion symmetries are preserved. As we mentioned above, in the charge ordering model for Fe_3_O_4_, each position on 16*d* sites has a face-centered translational symmetry, which is preserved. However, the <111> − direction threefold rotational symmetries are broken as shown in Fig. 1 (D and E).

ELC nematic phases have been widely known for their importance in distinguishing electronic structures and lattice from symmetry perspective with electron-driven mechanism, hence providing a unique way of probing the structure-property relationships in correlated materials (*34*). To date, ELC nematic phases are observed in cuprates (*35*), iron pnictides, manganites, and ruthenate, etc. (*33*), and those findings have generated far-reaching impacts in the research area. In report (*36*), the breaking of rotational symmetry was detected in copper oxide superconductors La_2−*x*_Sr*_x_*CuO_4_ via in-plane anisotropic electron transport. In addition, the direction of the electronic nematic order is detached from the lattice symmetry, which indicates that the nematicity is not reduced by lattice distortion. All the observations are very similar to ours. However, unlike most found ELC phases with rotational symmetry breaking from fourfold (C_4_) to twofold (C_2_), the present ELC phase in magnetite breaks the *O_h_* point group down to *D_3d_*, which breaks a different set of threefold rotational symmetry (for detailed analysis about the point group on the charge ordering model, see Supplementary Materials). After revisiting the original definition of nematic phases in classical liquid crystals, we conclude that the charge order in Fe_3_O_4_ is an unconventional type of electronic nematicity ever reported in correlated materials based on the following analysis. In classic liquid crystals, the disordered phase preserves the continuous three-dimensional rotational symmetry [SO(3)] and nematicity is characterized by a director (unit vector) order parameter, i.e., a traceless symmetric matrix with five independent components (*37*, *38*). In a cubic lattice with *O_h_* point group symmetry, this 5D representation splits into a 2D E_g_ representation and a 3D T_2g_ representation. The two components in the E_g_ representation characterize the C_4_ to C_2_ symmetry breaking, matching the observations in all other reported cases. In contrast, the T_2g_ representation with three components that breaks the *O_h_* symmetry down to *D_3d_* are the nematic order parameters, i.e., electronic order parameter, observed here for Fe_3_O_4_, which notably broadens the existence of electronic nematicity in electron-correlated materials and provides an advanced pathway to study the entanglement between the electrons and the lattice.

It is worthwhile to highlight that this nematic signature is observed above the metal-insulator transition temperature (*T* > *T*_v_), where charge carriers remain mobile. Because conventional charge order is expected to be washed away by mobile charge carriers, this observation suggests that the electronic nematicity plays a crucial role in the high-temperature phase. This is consistent with neutron scattering results (*7*, *10*, *11*), which does not observe lattice distortion or any building blocks of directional nature.

### {200} reflection intensity evolution upon cooling and warming

To further demonstrate the role of this charge order, or electronic nematicity, in the Verwey transition, we measured the intensity variation as a function of temperature during in situ cooling and heating experiments on Fe_3_O_4_ single crystals. Typical electron diffraction patterns above and below *T*_v_ are shown in Fig. 2A. The {200} reflections are denoted as the charge-ordered (CO) peaks at 300 K, while the SL reflections, i.e., forbidden reflections, are consistent with the monoclinic structure with trimerons below *T*_v_. Figure 2B shows the intensity measurements of the CO and SL peaks, and the measurements from the two samples are very similar. The CO peak intensity reflects the amplitude of the nematic order parameter. (The intensity of CO reflection as a function of valence charge on octahedral Fe sites is calculated and shown in fig. S10; the larger the charge disproportion, the higher the peak intensity.) The nematic signature remains nonzero above *T*_v_, even up to room temperature (RT). As the temperature decreases toward *T*_v_, a rapid increasing of the nematic order parameter is observed below 170 K. This temperature dependence invites comparisons to the nematic order observed in the high-temperature superconductor YBa_2_Cu_3_O_6+*x*_ (*33*, *39*).

In addition, peak width as a function of temperature measured from two samples is shown in Fig. 2C. The line profiles at a few typical temperatures are presented in Fig. 2D. The sharp CO reflections at all the temperatures demonstrate that the charge ordering phase is a long-range order. According to our measurements of the peak width, the coherent length of the nematic order is about 7.8 nm at 295 K and gradually increases to a value about 10.7 nm at 157 K in sample 1. Sample 2 displays a similar temperature evolution with slightly larger coherent lengths upon cooling. Right at *T*_v_, strong SL reflections appear and their intensity increases as the temperature cools down. Note that the CO peaks are coincided with the SL reflections in the low-temperature phase; thus, it is difficult to separate them in static observations. The results manifest an interplay of the electronic nematicity and the lattice order through thermal processes, which, on one hand, confirms the mechanism of charge order–induced transition in this material. When the sample was heated up from RT to higher temperatures, the {200} peak intensity got reduced and the peak width got broader. Because the sample was damaged above 423 K, we deduce that the hypothetical temperature at which the nematicity disappears is higher than 423 K and does not necessarily exist at all. The electron diffraction patterns taken during the heating process and the corresponding measurement results are shown in fig. S11.

The evolution from electronic nematicity to trimerons is a topic of interest in condensed matter physics because it provides insights into the underlying mechanisms that drive the formation of complex electronic phases in materials. For example, electronic nematicity (rhombohedral; cubic symmetry is already broken) can help to stabilize the trimeron order (with a monoclinic supercell) by providing a preferred directionality for the electronic degrees of freedom that participate in trimeron formation. Furthermore, the discovery of the electronic nematicity reveals that the Verwey transition belongs to a new universality class based on theoretical studies of ELC phase transitions, different from the conventional picture (see details in the “Two possible scenarios in the Verwey transition upon cooling” section in the Supplementary Materials).

### Transition from electronic nematicity to trimerons

We find interesting relationships that bridge the charge arrangements in electronic nematicity above *T*_v_ and the trimeron order below *T*_v_ via electron-phonon coupling. Because four variants of electronic nematicity exist equally, there can be twin structures with an order as spatial distribution or a random mixture along the ***c*** axis in a local volume. Note that two phonon modes Δ_5_ and X_3_ with their wave vectors *k*_**Δ**_ = (0, 0, 1/2) and *k*_**X**_ = (0, 0, 1) in cubic notation, respectively, were considered to play substantial roles in the structural phase transition at *T*_v_ (*7*, *10*, *40*, *41*). Since the ***c*** axis is doubled from cubic phase to monoclinic phase, the Δ_5_ mode with a (0, 0, 1/2) wave vector may help to regulate the stacking order of electronic nematicity along the ***c*** direction during the formation of trimeron order. Meanwhile, the X_3_ mode [together with other possible phonon modes, such as polaron tunneling (*42*, *43*)] in its wavelength may destroy the sheet-like charge ordering in electronic nematicity and create the linkage of charged atoms between the sheets to be trimeron orders. The atomic layers of octahedral Fe atoms with different valence in unit-cell configuration of the cubic and monoclinic phases are shown in figs. S13 and S14. The present findings bestow more character to the Δ_5_ and X_3_ phonon modes, which were initially selected on the basis of the symmetry breaking elements in lattice through the transition (*40*, *41*), suggesting that they could be essential to transform a nematic phase in electron substructure into the 3D trimeron order. To figure out how these phonon modes assist the dimensional switch of the electronic order during Verwey transition, MeV ultrafast electron diffraction (UED) method was used to detect the dynamic behavior of electron and phonon modes in the far-from-equilibrium state.

### UED study on the electron-phonon coupling in Fe_3_O_4_

**Fig. 3.**
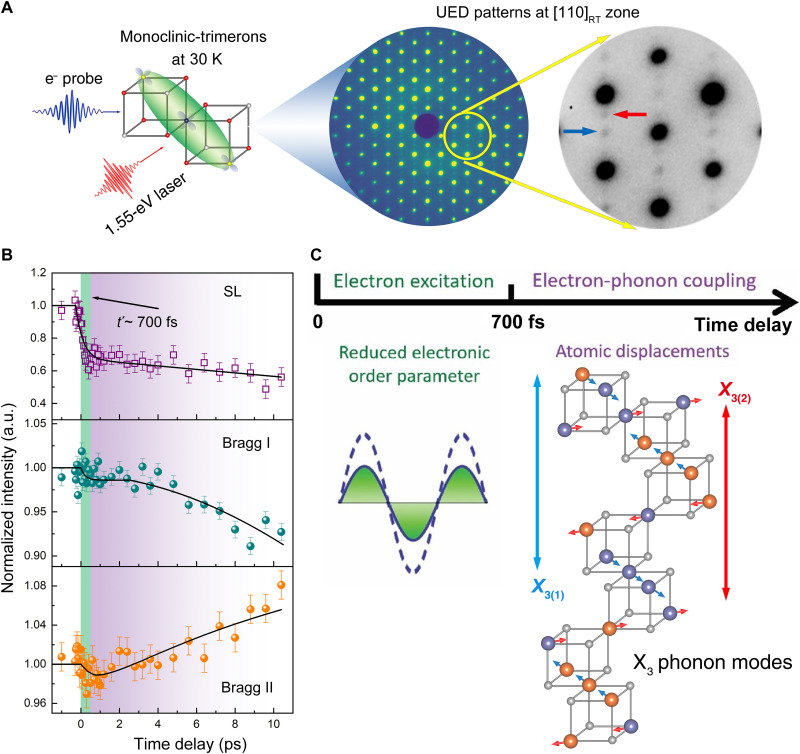
Ultrafast observations of dynamic processes in photoexcited Fe_3_O_4_. (**A**) Schematic diagram showing 1.55-eV laser as a pump and MeV electron beam as a probe. The UED sample is from sample 1. The UED patterns were obtained at *T* ~ 30 K along the [110] zone axis. Arrows in the enlarged pattern indicate the SL reflections from the monoclinic structure with the trimeron order. (**B**) Intensity variations of SL peaks and Bragg peaks as a function of delay time. Measurements from all the Bragg peaks show two distinct behaviors: one with intensity increase and others with intensity decrease as time goes by in a few picoseconds. Error bars represent the SD in the mean of intensity before time zero. The solid line is a guide to the eye. (**C**) Two-time regimes of the dynamic process were observed. One is the first 700 fs, labeled using *t*′, in which the electron excitation and thermalization are dominant, resulting in a reduced charge order parameter in the system. In the subsequent second time regime, lattice responds to the reduction of electronic order parameter by atomic displacement. Specifically, analysis indicates that atomic displacements are consistent with X_3_ phonon modes, shown in the crystal model with arrows pointing to the directions of atomic displacements.

Direct observations of the coupling between charge and specific phonon modes can be obtained from MeV UED results at the temperature ~30 K where the structure is monoclinic with the trimeron charge ordering. In the optical conductivity measurements, three main features at 0.6, 2.0, and 5 eV are related to intersite transitions between octahedral-site Fe ions, octahedral and tetrahedral-sites Fe ions, and charge transfer between O 2*p* and Fe 3*d* states, respectively, which has been confirmed by density functional theory calculations (*44*–*46*). In our UED experiment, the laser pulses (1.55 eV) are used to tune the charges between the octahedral site Fe ions in trimerons, which quenches the ordering phase, excites the structural lattice, and partially drives the system from the low-temperature phase to high-temperature phase, as suggested in (*43*, *47*, *48*). Figure 3A shows the experimental setup of the excitation and observation processes, i.e., a pump-probe scheme. Because the Bragg peaks are more sensitive to the lattice structure, we prepared a UED single-crystal sample at the [110] zone axis for intensity measurements from more Bragg/SL peaks than the [001] zone shown in Figs. 1 and 2. The intensity variation as a function of delay time is plotted in Fig. 3B, which gives two time scales during dynamic processes. The fast one takes place in the first 700 fs in which all the SL peak intensities drop quickly, while no obvious change can be measured from Bragg peak intensities. A slow dynamic process follows, which starts from ~700 fs and lasts at least a few picoseconds in our observations, which is consistent with the timescale observed in the ultrafast x-ray diffraction study (*47*). During this period, only minor changes were observed from SL peaks, while both increase and decrease in intensities were observed from Bragg peaks. Additional intensity measurements for the Bragg peaks can be found in the Supplementary Materials. From the intensity variation in the time domain as shown in Fig. 3B and fig. S16, we found that there is no observable oscillation behavior, implying that the photoexcited states are not induced by any coherent phonon modes (*49*, *50*). The absence of coherent modes may rely on multiple factors, e.g., pump energy, pump fluence, probe energy, etc. (*43*, *45*).

To understand the origin of the peak intensity variations, we performed dynamic electron diffraction analysis considering atomic charge distribution, atomic displacements, and existence of twin variants (see tables S4 and S5 about the twin variants and corresponding reflections in the Supplementary Materials). We calculated intensities of Bragg peaks and SL peaks by changing the valence electron on the octahedral sites in the monoclinic phase in fig. S17. The calculation indicates that the SL reflections are much more sensitive to the charge states, while the Bragg peaks are more sensitive to the atomic displacements (see figs. S19 and S20). The diffraction calculations reveal that the intensity change of the SL peaks mainly comes from the change in electronic order parameter, and the intensity variations of the Bragg peaks can be mostly attributed to atomic displacement, i.e., lattice distortions. As illustrated in Fig. 3C, during the delay time range in our experiments, electrons are excited and thermalized to reduce the electronic order parameter in trimerons in the first 700 fs after photoexcitation.

In the following a few picoseconds, lattice responds with atomic displacements from the initial monoclinic structure. In this step, we tested the effects of atomic displacements based on the Δ_5_, X_3_, and a few other frozen phonon modes that researchers often consider in this material (*51*, *52*) and find that atomic displacements based on only the X_3_ phonon mode (Fig. 3C) can provide consistent intensity variation with the experimental results (the intensity variations induced by the atomic displacements following different phonon modes are discussed in Materials and Methods and the Supplementary Materials). Thus, we conclude that, during the course of photoexcitation and relaxation that is different from the thermal process, the X_3_ phonon mode was preferably excited after the electron excitation at least with much more intensity responses than other phonon modes reflected in our UED measurement. As we mentioned above, the intensity variation without the coherent oscillation indicates that the photoexcited X_3_ phonon mode revealed using our experimental condition is likely not a coherent phonon and suggests that the change in the electron-electron interaction modifies the potential well of the X_3_ phonon mode during the photoexcitation process as mentioned in (*53*). Namely, the coupling between electrons and X_3_ phonon modes is stronger than other electron-phonon couplings in Fe_3_O_4_ crystals. This finding implies that X_3_ phonon modes play a major role in the nematic phase–trimeron phase transformation, which is responsible for regulating the charge transfer among the Fe ions in the layers and among layers. Moreover, first-principles calculations indicate that the X_3_ phonon mode is also in charge of the electric properties in Fe_3_O_4_—being an insulator by opening a gap at the Fermi surface during the phase transition (*9*, *41*, *54*).

What is more, the mechanisms of Verwey transition have been enormously debated for decades (*5*). The electron, phonon degrees of freedom and their interaction are the central topic, e.g., the electron-phonon coupling (*53*), order-disorder phase transition (*55*), and electron-electron interaction (*56*) are proposed to be crucial for Verwey transition. To figure out the transition mechanisms, various ultrafast methods have been used to explore dynamic behaviors of electronic structure and lattice structure. For example, using the time-resolved optical reflectivity measurements, the metallic and insulating phase separation was observed during a specific pump fluence regime in 1.55-eV laser pulses as shown in (*47*, *48*). In (*45*), it was shown that the ultrashort laser pulses with 3.1-eV generated symmetry-forbidden phonon modes, which is related to the critical fluctuations of electronic order due to the electron-phonon couplings. In our UED observations, we focused on the electron-phonon interactions and provided more direct evidence by taking advantage of electron sensitivities to both valence charge and lattice (*57*). We found that the X_3_ phonon mode was pinpointed during the photoexcitation process through the electron-phonon coupling, which was proposed in the theoretical studies (*40*, *41*). We conclude that the intimate coupling between charge and the phonon mode plays a vital role in the Verwey transition, which supports the mechanism of electron-phonon coupling in the debate. Last but not least, the X_3_ phonon excitation in dynamic observations may imply a manipulation of the electric properties of Fe_3_O_4_ in a short time range of a few picoseconds.

## DISCUSSION

In summary, the discovery of the unexpected symmetry breaking in the electronic state of Fe_3_O_4_ crystals at the temperature range far above *T*_v_ opens a window to the further works, including theoretical interpretation of the nature of the unconventional type of electronic nematicity and its role in the correlated materials. In addition, the findings here advance the understanding of the Verwey transition: It suggests that the high-temperature, charge ordering phase contributes to the phase transition. In the absence of the additional measurements, e.g., applying strain control, it is not possible to explicitly rule out the lattice distortion contribution to the symmetry breaking, although all the existing measurements indicate a role for electronic nematic order at the temperatures above *T*_v_. The findings described in this report connect the electronic nematicity order in the cubic lattice to the trimeron order in the monoclinic lattice through electron-phonon coupling, offering a transformative interpretation of the Verwey transition mechanism and innovative insights into the origin of the trimerons formation. The MeV UED pump-probe experiments not only provide a means to directly observe electron-phonon coupling through the dynamic process but also suggest a possible manipulation of the materials’ electric property by a specific phonon excitation using ultrafast laser pulses.

## MATERIALS AND METHODS

### Sample preparation

We chose Fe_3_O_4_ bulk single crystals from two resources, one is a mineral procured from SurfaceNet GmbH (sample 1) and the other is a laboratory synthesized crystal, which was grown at the Max Planck Institute for chemical physics of solids in Dresden (sample 2). Resistance measurements show that both crystals have a first-order MIT, and the transition temperature *T*_v_ ~ 115 K for sample 1 and *T*_v_ ~ 123.4 K for sample 2 (see the Supplementary Materials). The UED sample was cut from sample 1 and prepared using mechanical polishing and focused ion beam and thinned down to electron transparent.

### MeV UED

The experiments were performed on the MeV-UED (*58*, *59*) setup at Stanford Linear Accelerator Laboratory, which can achieve <150-fs [full width at half maximum (FWHM)] temporal resolution. Using this experimental setup, the sample was excited by a 75-fs (FWHM) laser pulse with a photon energy of 1.55 eV and a fluence of 4 mJ/cm^2^. The optical pulses at a repetition rate of 180 Hz were focused on the sample. The laser pump pulse and probing electron beam are colinear in the UED instrument. The sample was excited by synchronized 3.5-MeV electron pulses containing ~10^6^ electrons with an illuminated area of 100 μm in diameter. The sample temperature of 30 K was achieved using a conducting sample holder by liquid helium.

### KeV transmission electron diffraction

The diffraction patterns shown in Figs. 1B and 2A were obtained from a JEOL ARM 200F microscope operating at 200 kV at Brookhaven National Laboratory. In situ cooling TEM experiment was carried out using the Gatan liquid nitrogen cooling stage with double tilt capability. The experiments were carried out at the lowest attainable temperature of ~90 K. Two methods were used to prepare the samples: (i) crushing the sample into pieces and suspended on the TEM Cu grid-coated with carbon thin film and (ii) using focused ion beam.

### Electron diffraction simulations for TEM and UED data

To understand the origin of the intensity variation in the electron diffraction patterns, we performed dynamic diffraction simulation based on the Bloch wave method using the computer code developed in-house. See the Supplementary Materials for the dynamic simulation.

## Supplementary Materials

### This PDF file includes:

Supplementary Text Figs. S1 to S20 Tables S1 to S5 References

## References

[1] E. J. W. Verwey, Electronic conduction of magnetite (Fe_3_O_4_) and its transition point at low temperatures. Nature 144, 327–328 (1939).

[2] M. S. Senn, J. P. Wright, J. P. Attfield, Charge order and three-site distortions in the Verwey structure of magnetite. Nature 481, 173–176 (2012). 10.1038/nature10704 22190035

[3] E. J. W. Verwey, P. W. Haayman, Electronic conductivity and transition point of magnetite (“Fe_3_O_4_”). Physica 8, 979–987 (1941).

[4] M. Coey, Charge-ordering in oxides. Nature 430, 155–157 (2004). 15241400 10.1038/430155a

[5] F. Walz, The Verwey transition - a topical review. J. Phys. Condens. Matter 14, R285–R340 (2002).

[6] J. García, G. Subías, The Verwey transition—A new perspective. J. Phys. Condens. Matter 16, R145–R178 (2004).

[7] Y. Fujii, G. Shirane, Y. Yamada, Study of the 123-K phase transition of magnetite by critical neutron scattering. Phys. Rev. B. 11, 2036–2041 (1975).

[8] K. Siratori, Y. Ishii, Y. Morii, S. Funahashi, S. Todo, A. Yanase, Neutron diffuse scattering study of the high temperature phase of Fe_3_O_4_- I, determination of atomic displacements at the *X* point in the Brillouin zone. J. Phys. Soc. Jpn 67, 2818–2827 (1998).

[9] H.-T. Jeng, G. Y. Guo, D. J. Huang, Charge-orbital ordering and Verwey transition in magnetite. Phys. Rev. Lett. 93, 156403 (2004). 15524911 10.1103/PhysRevLett.93.156403

[10] S. M. Shapiro, M. Iizumi, G. Shirane, Neutron scattering study of the diffuse critical scattering associated with the Verwey transition in magnetite Fe_3_O_4_. Phys. Rev. B 14, 200–207 (1976).

[11] Y. Yamada, N. Wakabayashi, R. M. Nicklow, Neutron diffuse scattering in magnetite due to molecular polarons. Phys. Rev. B 21, 4642–4648 (1980).

[12] A. Bosak, D. Chernyshov, M. Hoesch, P. Piekarz, M. Le Tacon, M. Krisch, A. Kozłowski, A. M. Oleś, K. Parlinski, Short-range correlations in magnetite above the Verwey temperature. Phys. Rev. X 4, 011040 (2014).

[13] G. Perversi, E. Pachoud, J. Cumby, J. M. Hudspeth, J. P. Wright, S. A. J. Kimber, J. Paul Attfield, Co-emergence of magnetic order and structural fluctuations in magnetite. Nat. Commun. 10, 2857 (2019). 31253806 10.1038/s41467-019-10949-9 PMC6599026

[14] H. Elnaggar, R. Wang, S. Lafuerza, E. Paris, A. C. Komarek, H. Guo, Y. Tseng, D. McNally, F. Frati, M. W. Haverkort, M. Sikora, T. Schmitt, F. M. F. de Groot, Possible absence of trimeron correlations above the Verwey temperature in Fe_3_O_4_. Phys. Rev. B 101, 085107 (2020).

[15] P. W. Anderson, Ordering and Antiferromagnetism in Ferrites. Phys. Rev. 102, 1008–1013 (1956).

[16] R. Aragón, R. J. Rasmussen, J. P. Shepherd, J. W. Koenitzer, J. M. Honig, Effect of stoichiometry changes on electrical properties of magnetite. J. Magn. Magn. Mater. 54-57, 1335–1336 (1986).

[17] Z. Kakol, J. M. Honig, Influence of deviations from ideal stoichiometry on the anisotropy parameters of magnetite Fe_3(1−δ)_O_4_. Phys. Rev. B 40, 9090–9097 (1989). 10.1103/physrevb.40.9090 9991395

[18] A. Chainani, T. Yokoya, T. Morimoto, T. Takahashi, S. Todo, High-resolution photoemission spectroscopy of the Verwey transition in Fe_3_O_4_. Phys. Rev. B 51, 17976–17979 (1995). 10.1103/physrevb.51.17976 9978834

[19] D. Schrupp, M. Sing, M. Tsunekawa, H. Fujiwara, S. Kasai, A. Sekiyama, S. Suga, T. Muro, V. A. M. Brabers, R. Claessen, High-energy photoemission on Fe_3_O_4_: Small polaron physics and the Verwey transition. Europhys. Lett. 70, 789–795 (2005).

[20] T. K. Shimizu, J. Jung, H. S. Kato, Y. Kim, M. Kawai, Termination and Verwey transition of the (111) surface of magnetite studied by scanning tunneling microscopy and first-principles calculations. Phys. Rev. B 81, 235429 (2010).

[21] W. H. Bragg, The Structure of Magnetite and the Spinels. Nature 95, 561–561 (1915).

[22] M. E. Fleet, The structure of magnetite. Acta Crystallogr. B 37, 917–920 (1981).

[23] J.-H. Chu, H.-H. Kuo, J. G. Analytis, I. R. Fisher, Divergent nematic susceptibility in an iron arsenide superconductor. Science 337, 710–712 (2012). 22879513 10.1126/science.1221713

[24] P. Malinowski, Q. Jiang, J. J. Sanchez, J. Mutch, Z. Liu, P. Went, J. Liu, P. J. Ryan, J.-W. Kim, J.-H. Chu, Suppression of superconductivity by anisotropic strain near a nematic quantum critical point. Nat. Phys. 16, 1189–1193 (2020).

[25] A. E. Böhmer, P. Burger, F. Hardy, T. Wolf, P. Schweiss, R. Fromknecht, M. Reinecker, W. Schranz, C. Meingast, Nematic susceptibility of hole-doped and electron-doped BaFe_2_As_2_ iron-based superconductors from shear modulus measurements. Phys. Rev. Lett. 112, 047001 (2014). 24580480 10.1103/PhysRevLett.112.047001

[26] S. Borroni, J. Teyssier, P. Piekarz, A. B. Kuzmenko, A. M. Oleś, J. Lorenzana, F. Carbone, Light scattering from the critical modes of the Verwey transition in magnetite. Phys. Rev. B 98, 184301 (2018).

[27] J. García, G. Subías, M. G. Proietti, H. Renevier, Y. Joly, J. L. Hodeau, J. Blasco, M. C. Sánchez, J. F. Bérar, Resonant “Forbidden” Reflections in Magnetite. Phys. Rev. Lett. 85, 578–581 (2000). 10991344 10.1103/PhysRevLett.85.578

[28] T. Hahn, *Volume A: Space Group Symmetry* in *International Tables for Crystallography*, T. Hahn, Ed. (Springer) (2002); https://link.springer.com/referencework/10.1107/97809553602060000100).

[29] Y. Zhu, L. Wu, J. Tafto, Accurate measurements of valence electron distribution and interfacial lattice displacement using quantitative electron diffraction. Microsc. Microanal. 9, 442–456 (2003). 19771700 10.1017/s143192760303037x

[30] J. Fink, E. Schierle, E. Weschke, J. Geck, Resonant elastic soft x-ray scattering. Rep. Prog. Phys. 76, 056502 (2013). 23563216 10.1088/0034-4885/76/5/056502

[31] J. García, G. Subías, M. G. Proietti, J. Blasco, H. Renevier, J. L. Hodeau, Y. Joly, Absence of charge ordering below the Verwey transition temperature in magnetite. Phys. Rev. B 63, 054110 (2001).

[32] G. Subías, J. García, J. Blasco, J. Herrero-Martín, M. C. Sánchez, J. Orna, L. Morellón, Structural distortion, charge modulation and local anisotropies in magnetite below the Verwey transition using resonant X-ray scattering. J. Synchrotron Radiat. 19, 159–173 (2012). 22338674 10.1107/S0909049512001367

[33] E. Fradkin, S. A. Kivelson, M. J. Lawler, J. P. Eisenstein, A. P. Mackenzie, Nematic fermi fluids in condensed matter physics. Annu. Rev. Condens. Matter Phys. 1, 153–178 (2010).

[34] S. A. Kivelson, E. Fradkin, V. J. Emery, Electronic liquid-crystal phases of a doped Mott insulator. Nature 393, 550–553 (1998).

[35] J. M. Tranquada, B. J. Sternlieb, J. D. Axe, Y. Nakamura, S. Uchida, Evidence for stripe correlations of spins and holes in copper oxide superconductors. Nature 375, 561–563 (1995).

[36] J. Wu, A. T. Bollinger, X. He, I. Božović, Spontaneous breaking of rotational symmetry in copper oxide superconductors. Nature 547, 432–435 (2017). 28748933 10.1038/nature23290

[37] P. M. Chaikin, T. C. Lubensky, *Principles of Condensed Matter Physics* (Cambridge Univ. Press, 2012;www.cambridge.org/core/books/principles-of-condensed-matter-physics/70C3D677A9B5BEC4A77CBBD0A8A23E64).

[38] P. G. de Gennes, J. Prost, *The Physics of Liquid Crystals* (Clarendon Press, 1993).

[39] E. Fradkin, S. A. Kivelson, J. M. Tranquada, Colloquium: Theory of intertwined orders in high temperature superconductors. Rev. Mod. Phys. 87, 457–482 (2015).

[40] P. Piekarz, K. Parlinski, A. M. Oleś, Mechanism of the Verwey transition in magnetite. Phys. Rev. Lett. 97, 156402 (2006). 17155347 10.1103/PhysRevLett.97.156402

[41] P. Piekarz, K. Parlinski, A. M. Oleś, Origin of the Verwey transition in magnetite: Group theory, electronic structure, and lattice dynamics study. Phys. Rev. B 76, 165124 (2007).

[42] K. Yamauchi, T. Fukushima, S. Picozzi, Ferroelectricity in multiferroic magnetite Fe_3_O_4_ driven by noncentrosymmetric Fe^2+^/Fe^3+^ charge-ordering: First-principles study. Phys. Rev. B 79, 212404 (2009).

[43] E. Baldini, C. A. Belvin, M. Rodriguez-Vega, I. O. Ozel, D. Legut, A. Kozłowski, A. M. Oleś, K. Parlinski, P. Piekarz, J. Lorenzana, G. A. Fiete, N. Gedik, Discovery of the soft electronic modes of the trimeron order in magnetite. Nat. Phys. 16, 541–545 (2020).

[44] S. K. Park, T. Ishikawa, Y. Tokura, Charge-gap formation upon the Verwey transition in Fe_3_O_4_. Phys. Rev. B 58, 3717–3720 (1998).

[45] S. Borroni, E. Baldini, V. M. Katukuri, A. Mann, K. Parlinski, D. Legut, C. Arrell, F. van Mourik, J. Teyssier, A. Kozlowski, P. Piekarz, O. V. Yazyev, A. M. Oleś, J. Lorenzana, F. Carbone, Coherent generation of symmetry-forbidden phonons by light-induced electron-phonon interactions in magnetite. Phys. Rev. B 96, 104308 (2017).

[46] I. Leonov, A. N. Yaresko, V. N. Antonov, V. I. Anisimov, Electronic structure of charge-ordered Fe_3_O_4_ from calculated optical, magneto-optical Kerr effect, and OK-edge x-ray absorption spectra. Phys. Rev. B 74, 165117 (2006).

[47] S. de Jong, R. Kukreja, C. Trabant, N. Pontius, C. F. Chang, T. Kachel, M. Beye, F. Sorgenfrei, C. H. Back, B. Bräuer, W. F. Schlotter, J. J. Turner, O. Krupin, M. Doehler, D. Zhu, M. A. Hossain, A. O. Scherz, D. Fausti, F. Novelli, M. Esposito, W. S. Lee, Y. D. Chuang, D. H. Lu, R. G. Moore, M. Yi, M. Trigo, P. Kirchmann, L. Pathey, M. S. Golden, M. Buchholz, P. Metcalf, F. Parmigiani, W. Wurth, A. Föhlisch, C. Schüßler-Langeheine, H. A. Dürr, Speed limit of the insulator–metal transition in magnetite. Nat. Mater. 12, 882–886 (2013). 23892787 10.1038/nmat3718

[48] F. Randi, I. Vergara, F. Novelli, M. Esposito, M. Dell’Angela, V. A. M. Brabers, P. Metcalf, R. Kukreja, H. A. Dürr, D. Fausti, M. Grüninger, F. Parmigiani, Phase separation in the nonequilibrium Verwey transition in magnetite. Phys. Rev. B 93, 054305 (2016).

[49] K. Sokolowski-Tinten, C. Blome, J. Blums, A. Cavalleri, C. Dietrich, A. Tarasevitch, I. Uschmann, E. Förster, M. Kammler, M. Horn-von-Hoegen, D. von der Linde, Femtosecond X-ray measurement of coherent lattice vibrations near the Lindemann stability limit. Nature 422, 287–289 (2003). 12646915 10.1038/nature01490

[50] S. Gerber, S.-L. Yang, D. Zhu, H. Soifer, J. A. Sobota, S. Rebec, J. J. Lee, T. Jia, B. Moritz, C. Jia, A. Gauthier, Y. Li, D. Leuenberger, Y. Zhang, L. Chaix, W. Li, H. Jang, J.-S. Lee, M. Yi, G. L. Dakovski, S. Song, J. M. Glownia, S. Nelson, K. W. Kim, Y.-D. Chuang, Z. Hussain, R. G. Moore, T. P. Devereaux, W.-S. Lee, P. S. Kirchmann, Z.-X. Shen, Femtosecond electron-phonon lock-in by photoemission and x-ray free-electron laser. Science 357, 71–75 (2017). 28684521 10.1126/science.aak9946

[51] M. Iizumi, T. F. Koetzle, G. Shirane, S. Chikazumi, M. Matsui, S. Todo, Structure of magnetite (Fe_3_O_4_) below the Verwey transition temperature. Acta Crystallogr. B 38, 2121–2133 (1982).

[52] J. P. Wright, J. P. Attfield, P. G. Radaelli, Charge ordered structure of magnetite Fe_3_O_4_ below the Verwey transition. Phys. Rev. B 66, 214422 (2002).

[53] M. Hoesch, P. Piekarz, A. Bosak, M. Le Tacon, M. Krisch, A. Kozłowski, A. M. Oleś, K. Parlinski, Anharmonicity due to electron-phonon coupling in magnetite. Phys. Rev. Lett. 110, 207204 (2013). 25167445 10.1103/PhysRevLett.110.207204

[54] H. P. Pinto, S. D. Elliott, Mechanism of the Verwey transition in magnetite: Jahn–Teller distortion and charge ordering patterns. J. Phys. Condens. Matter 18, 10427–10436 (2006). 21690927 10.1088/0953-8984/18/46/010

[55] S. Borroni, G. S. Tucker, F. Pennacchio, J. Rajeswari, U. Stuhr, A. Pisoni, J. Lorenzana, H. M. Rønnow, F. Carbone, Mapping the lattice dynamical anomaly of the order parameters across the Verwey transition in magnetite. New J. Phys. 19, 103013 (2017).

[56] B. Lorenz, D. Ihle, Electron–phonon versus coulomb interaction effects at the Verwey transition of Fe_3_O_4_. Phys. Status Solidi B. 96, 659–669 (1979).

[57] J. Li, J. Li, K. Sun, L. Wu, R. Li, J. Yang, X. Shen, X. Wang, H. Luo, R. J. Cava, I. K. Robinson, X. Jin, W. Yin, Y. Zhu, J. Tao, Concurrent probing of electron-lattice dephasing induced by photoexcitation in 1T-TaSeTe using ultrafast electron diffraction. Phys. Rev. B 101, 100304 (2020).

[58] P. Zhu, Y. Zhu, Y. Hidaka, L. Wu, J. Cao, H. Berger, J. Geck, R. Kraus, S. Pjerov, Y. Shen, R. I. Tobey, J. P. Hill, X. J. Wang, Femtosecond time-resolved MeV electron diffraction. New J. Phys. 17, 063004 (2015).

[59] S. P. Weathersby, G. Brown, M. Centurion, T. F. Chase, R. Coffee, J. Corbett, J. P. Eichner, J. C. Frisch, A. R. Fry, M. Gühr, N. Hartmann, C. Hast, R. Hettel, R. K. Jobe, E. N. Jongewaard, J. R. Lewandowski, R. K. Li, A. M. Lindenberg, I. Makasyuk, J. E. May, D. McCormick, M. N. Nguyen, A. H. Reid, X. Shen, K. Sokolowski-Tinten, T. Vecchione, S. L. Vetter, J. Wu, J. Yang, H. A. Dürr, X. J. Wang, Mega-electron-volt ultrafast electron diffraction at SLAC national accelerator laboratory. Rev. Sci. Instrum. 86, 073702 (2015). 26233391 10.1063/1.4926994

[60] E. J. Kirkland, *Advanced Computing in Electron Microscopy* (Springer International Publishing, Cham, 2020; 10.1007/978-3-030-33260-0).

[61] J. M. Zuo, J. Pacaud, R. Hoier, J. C. H. Spence, Experimental measurement of electron diffuse scattering in magnetite using energy-filter and imaging plates. Micron 31, 527–532 (2000). 10831297 10.1016/s0968-4328(99)00133-x

[62] P. G. de Gennes, An analogy between superconductors and smectics A. Solid State Commun. 10, 753–756 (1972).

[63] B. I. Halperin, T. C. Lubensky, S. Ma, First-order phase transitions in superconductors and Smectic-A liquid crystals. Phys. Rev. Lett. 32, 292–295 (1974).

