# Supplementary material for: Verwey transition as evolution from electronic nematicity to trimerons via electron-phonon coupling
Source: Sci Adv. 2023 Jun 9;9(23):eadf8220. doi: 10.1126/sciadv.adf8220 (PMC10256157; doi:10.1126/sciadv.adf8220)
Supplement: Supplementary file 1 — Supplementary Text Figs. S1 to S20 Tables S1 to S5 References [file sciadv.adf8220_sm.pdf]

Science Advances
AAAS

Supplementary Materials for

Verwey transition as evolution from electronic nematicity to trimerons via electron-phonon coupling

Wei Wang *et al.*

Corresponding author: Wei Wang, wangweipositron@outlook.com; Jing Tao, jingtao1975@gmail.com

Sci. Adv. **9**, eadf8220 (2023)
DOI: 10.1126/sciadv.adf8220

This PDF file includes:

Supplementary Text
Figs. S1 to S20
Tables S1 to S5
References

Supplementary Text

Electronic resistance measurement results

Since the Verwey transition is sensitive to the stoichiometry of the magnetite crystal, we chose two single crystals, i.e., Sample 1 and Sample 2, to perform the electron diffraction experiment. The resistance measurement result for these two single crystals is shown below, which demonstrate the high quality of the single crystals. According to the phase transition temperature measured from Fig. S1, we estimated the stoichiometries of these two crystals based on the measurement results in(*16*): Sample 1 $Fe_{3(1+0.0005)}O_4$ and Sample 2 $Fe_{3(1-0.001)}O_4$.

Electron diffraction patterns as a function of temperature

To examine the Verwey transition in the Sample 1 (commercial) and Sample 2 (lab grown), we cooled down the samples from room temperature (300 K) to 90 K, then started to warm up and the electron diffraction patterns were captured at different temperatures in the TEM, the results are shown in Figs. S2 and S3. When the sample is transformed from spinel cubic phase to monoclinic phase, the forbidden reflections in the high-temperature cubic phase appear at low temperature due to the symmetry breaking as shown in Fig. S2b. Most of the forbidden peaks disappear above T_v, e.g., shown in Fig. S2c, but a few reflections, e.g., {200} reflections enlarged in inserts, with low $\boldsymbol{q}$ values are remained up to high temperature, like at 423 K in Fig. S2f. Sample 2 shows the same feature and the *in situ* cooling TEM results are in Fig. S3.

Multiple scattering effect to the {200} reflections

To verify the formation of {200} reflections in the <001> direction are not induced by multiple scattering effect, we performed the dynamic electron diffraction simulation including an appropriate sample thickness and multiple scattering effect. The sample thickness is 60 nm used in the simulation, which will be further discussed in the next section. The dynamic diffraction simulation method is based on the Bloch wave method (*61*). According to the published standard crystal structure of cubic phase (*22*), the simulated result is shown in Fig. S4. The {200} reflections are still forbidden, indicating that the {200} reflections cannot arise from multiple scattering effect in the <001> orientated diffraction pattern.

Structure factors of {200} reflections in charge ordering models

The forbidden peaks observed in the experimental pattern indicate that a symmetry breaking exists in the cubic phase. To figure out the origin of the {200} reflection, we first consider the charge ordering case. The structure factor (*F*) for each reflection (*hkl*) in the diffraction is:

$$F(hkl) = \sum_{j=1}^{N} f_j \cdot \exp\left[2\pi i(hx_j + ky_j + lz_j)\right]$$

Where f_j is the atom form factor, x_j, y_j, z_j are the atomic coordinates in the unit cell, respectively. The Wyckoff position for each element in Fe_3O_4 in the cubic phase is shown in Table S1.

We presume that the {200} peaks are related to the Fe ions on 16*d* symmetry sites and there are only two types of valence state on Fe ions, i.e., $Fe^{(2.5+\delta1)+}$ and $Fe^{(2.5+\delta2)+}$. Additionally, considering the absence of other "forbidden" peaks shown in the monoclinic phase, we should preserve the face centered translational symmetry in the charge ordering model. Based on these conditions, we separate Fe ions on the octahedral sites into 4 subgroups with respect to valence state on Fe ions. Namely, the atom form factor of Fe ions in each subgroup would be f_i, i =1, 2, 3, 4.

According to the f.c.c symmetry in $Fd\bar{3}m$ space group, the equivalent atom positions of the octahedral Fe ions shown in Table S2.

$$\begin{aligned}\boldsymbol{F_{200}} = & f_1\cdot\exp[2\pi i\cdot 2\cdot 1/2] + f_1\cdot\exp[2\pi i\cdot 2\cdot 1/2] + f_1\cdot\exp[2\pi i\cdot 2\cdot 0] + f_1\cdot\exp[2\pi i\cdot 2\cdot 0] + \ldots \\ & f_2\cdot\exp[2\pi i\cdot 2\cdot 1/4] + f_2\cdot\exp[2\pi i\cdot 2\cdot 1/4] + f_2\cdot\exp[2\pi i\cdot 2\cdot 3/4] + f_2\cdot\exp[2\pi i\cdot 2\cdot 3/4] + \ldots \\ & f_3\cdot\exp[2\pi i\cdot 2\cdot 3/4] + f_3\cdot\exp[2\pi i\cdot 2\cdot 3/4] + f_3\cdot\exp[2\pi i\cdot 2\cdot 1/4] + f_3\cdot\exp[2\pi i\cdot 2\cdot 1/4] + \ldots \\ & f_4\cdot\exp[2\pi i\cdot 2\cdot 0] + f_4\cdot\exp[2\pi i\cdot 2\cdot 0] + f_4\cdot\exp[2\pi i\cdot 2\cdot 1/2] + f_4\cdot\exp[2\pi i\cdot 2\cdot 1/2] + \ldots \\ = & 4 f_1 - 4 f_2 - 4 f_3 + 4 f_4 = 4(f_1 - f_2 - f_3 + f_4)\end{aligned}$$

$$\begin{aligned}\boldsymbol{F_{020}} = & f_1\cdot\exp[2\pi i\cdot 2\cdot 1/2] + f_1\cdot\exp[2\pi i\cdot 2\cdot 0] + f_1\cdot\exp[2\pi i\cdot 2\cdot 1/2] + f_1\cdot\exp[2\pi i\cdot 2\cdot 0] + \ldots \\ & f_2\cdot\exp[2\pi i\cdot 2\cdot 3/4] + f_2\cdot\exp[2\pi i\cdot 2\cdot 1/4] + f_2\cdot\exp[2\pi i\cdot 2\cdot 3/4] + f_2\cdot\exp[2\pi i\cdot 2\cdot 1/4] + \ldots \\ & f_3\cdot\exp[2\pi i\cdot 2\cdot 0] + f_3\cdot\exp[2\pi i\cdot 2\cdot 1/2] + f_3\cdot\exp[2\pi i\cdot 2\cdot 0] + f_3\cdot\exp[2\pi i\cdot 2\cdot 1/2] + \ldots \\ & f_4\cdot\exp[2\pi i\cdot 2\cdot 1/4] + f_4\cdot\exp[2\pi i\cdot 2\cdot 3/4] + f_4\cdot\exp[2\pi i\cdot 2\cdot 1/4] + f_4\cdot\exp[2\pi i\cdot 2\cdot 3/4] + \ldots \\ = & 4 f_1 - 4 f_2 + 4 f_3 - 4 f_4 = 4(f_1 - f_2 + f_3 - f_4)\end{aligned}$$

The presence of the (±200) reflections indicate $f_1 + f_4 \neq f_2 + f_3$ and the presence of the (0±20) reflections indicate $f_1 + f_3 \neq f_2 + f_4$. To meet these conditions, one of the atom form factors should be different with the other three atom form factors, that is, the valence charge in one subgroup is different with other subgroups. For example, in subgroup 1 $Fe^{(2.5+\delta1)+}$ is Fe^{2+}, and subgroups 2, 3, 4, the $Fe^{(2.5+\delta2)+} = Fe^{2.76+}$ in order to keep the system neutral. Based on this charge ordering model, the originally forbidden {200} reflections can be observable due to the rotational symmetry breaking. There are 4 subgroups on the octahedral sites, each subgroup can be chosen to be different with the other three subgroups, therefore there are four twin variants in total. In each twin variant only one three-fold rotational symmetry along <111> direction is preserved, and the other three rotational symmetries are broken as shown in Fig. S5.

The dynamic electron diffraction simulation result is shown in Fig. S6. The intensity ratios of the reflections (I_{200}:I_{240}:I_{600}) in the experiment and simulation is summarized in Fig. 1g. The sample thickness for the simulation is determined to be 60 nm. The values of valence states on Fe ions are 2.54 and 2.486 for the simulation.

Structure factors of {200} reflections in atomic displacements models

With regards to atomic displacements model, the 16*d* Fe ions are separated in the same way as in the charge ordering model. That is, 16*d* site symmetry is broken, the face centered translational symmetry is preserved.

In the atomic displacements model shown in Table S3, we assume that there is no charge ordering on the Fe ions firstly. The atom form factor is f. Therefore, the structure factor of (200) reflection can be written:

$$\boldsymbol{F}_{200} = f\cdot\exp[2\pi i\cdot 2\cdot(1/2+\delta_1)] + f\cdot\exp[2\pi i\cdot 2\cdot(1/2+\delta_1)] + f\cdot\exp[2\pi i\cdot 2\cdot\delta_1] + f\cdot\exp[2\pi i\cdot 2\cdot\delta_1] + \ldots$$
$$f\cdot\exp[2\pi i\cdot 2\cdot(1/4+\delta_2)]+f\cdot\exp[2\pi i\cdot 2\cdot(1/4+\delta_2)]+ f\cdot\exp[2\pi i\cdot 2\cdot(3/4+\delta_2)] + f\cdot\exp[2\pi i\cdot 2\cdot(3/4+\delta_2)] + \ldots$$
$$f\cdot\exp[2\pi i\cdot 2\cdot(3/4+\delta_2)]+f\cdot\exp[2\pi i\cdot 2\cdot(3/4+\delta_2)] + f\cdot\exp[2\pi i\cdot 2\cdot(1/4+\delta_2)] + f\cdot\exp[2\pi i\cdot 2\cdot(1/4+\delta_2)] + \ldots$$
$$f\cdot\exp[2\pi i\cdot 2\cdot\delta_2] + f\cdot\exp[2\pi i\cdot 2\cdot\delta_2] + f\cdot\exp[2\pi i\cdot 2\cdot(1/2+\delta_2)] + f\cdot\exp[2\pi i\cdot 2\cdot(1/2+\delta_2)]$$
$$= 4 f\cdot[\exp(2\pi i\cdot 2\cdot\delta_1) - \exp(2\pi i\cdot 2\cdot\delta_2)]$$

To ensure $\boldsymbol{F}_{200}$ is different from zero, $\delta_1 \neq \delta_2$.

The dynamic electron diffraction simulation result is shown in Fig. S7. The atomic displacement is $0.005\boldsymbol{a_c}$ (~ 0.042 Å), which is larger than the spatial resolution of neutron scattering in(*7, 10*). However, the "forbidden" peaks, e.g., {200} and {600}, are not observed in the neutron scattering experiments, which indicate the extra "forbidden" peaks are mainly from electronic structure, i.e., charge ordering phase.

Space group and point group analysis based on the CO model

The point group of $Fd\bar{3}m$ space group is O_h, which has 48 symmetry operations. The charge ordering structure by splitting atomic coordinates on *16d* site reduces the symmetry operations from 48 to 12. According to the maximum non-isomorphic subgroups in $Fd\bar{3}m$ space group, the point group is reduced from O_h to D_{3d}, and the corresponding space group is transformed from $Fd\bar{3}m\ (No. 227)$ to $R\bar{3}m\ (No. 166)$. As we mentioned above, there are four types of charge distribution model. In each type of model, one three-fold rotational symmetry along <111> directions is preserved, the rotational symmetry in the other three directions are broken.

CO peak intensity and diffuse scattering intensity comparison

In the *in situ* cooling TEM experiment, we observed diffuse scattering signal right above phase transition temperature T_v. The diffraction pattern shown below in Fig. S8 is taken at 121 K from Sample 1. The sharp "forbidden" peaks from the monoclinic phase become smear and turn to diffuse. Simultaneously, the charge ordering peaks are still observable. The intensities of the diffuse scattering signal and charge ordering peaks are comparable. However, the neutron study in(*7, 10, 11*) detected the diffuse scattering signal but did not observe charge ordering peaks, which

indicates electronic ordering contributes more to the {200} reflections in the high-temperature cubic phase.

Form factor calculation for electron scattering and X-ray scattering

In order to compare the sensitivity to the valence electron on Fe ions, we calculated to the form factor Fe^{2+} and Fe^{3+} form factors as a function of scattering vector, the calculated form factor squared (f^2) are shown in Fig. S9. At the scattering vector of {200} reflection, $\boldsymbol{q}$ = 0.119 $Å^{-1}$, the f^2 difference between $f_e^2(Fe^{2+})$ and $f_e^2(Fe^{3+})$ is much larger than that between $f_x^2(Fe^{2+})$ and $f_x^2(Fe^{3+})$, which illustrates electron scattering is more sensitive to the valence charge disproportion at small scattering angles.

CO peak intensity as a function of valence charge on Fe ions

Figure 2b shows that the intensity of CO peak increases upon cooling and reaches a maximum value around the phase transition temperature. To figure out the intensity variation in the cubic phase, which is mainly related to the charge ordering, we calculated the intensity variations by changing the charge discrepancy on the Fe ions. The calculated result is shown in Fig. S10. When the charge is uniformly distributed on the Fe ions ($Fe_{oct.}^{2.5+}$), that is, no charge ordering exists (δ_1= δ_2 = 0), the CO peak intensity is zero. The CO peak shows up, when the charge discrepancy exists among the Fe ions shown in Fig. S10b. The calculated result also demonstrates that the larger charge discrepancy, the higher intensity on CO peak.

In situ heating TEM experiment

To explore whether the nematic phase can be thermally removed at high temperature, Sample 1 was gradually heated up from room temperature to 851 K and the electron diffraction patterns were captured at 325 K, 349 K, 372 K, 423 K, 625 K, 758 K and 851 K, respectively. The diffraction patterns at a few temperatures are shown in Figs. S11a-S11d. The CO peaks can be observed until 423 K and there are some extra peaks start to show up at 625 K. Additionally, the diffuse scattering signal is enhanced in the background at higher temperatures. we infer that the sample could be damaged by the heating effect, due to the sample being thin susceptible to stoichiometry changes at high temperatures. We analyzed the electron diffraction patterns taken below 625 K and the results are shown in Fig. S11e. Upon warming, the CO peak intensity gets reduced and the peak width gets broader, demonstrating the charge disproportion between Fe ions is smaller and the coherent length is decreased due to the thermal effect. Based on the *in situ* heating experiment, we deduce that the hypothetical temperature at which the nematicity disappears is higher than 423 K and does not necessarily exist at all.

Two possible scenarios in the Verwey transition upon cooling

The precursor behaviors in the Verwey transition have been observed and discussed, which presented as diffuse scattering in the diffraction techniques, such as, electron diffraction,

synchrotron x-ray diffraction and neutron scattering (*10–12*, *62*). The temperature range of the diffuse scattering observation highly depends on the sample quality and the stoichiometry. It is worthy of mentioning that we observed these diffuse scattering as well on the electron diffraction patterns from our well calibrated Fe_3O_4 samples: above the Verwey temperature, the diffuse scattering signals have significantly broadened and weak intensity distribution at barely defined $\boldsymbol{q}$ position similar (but not identical) to those reflections from the low-temperature trimeron phase. Hitherto, the origin of the diffuse scattering is still a highly debated issue that could arise from multiple possibilities, such as a short-range trimeron order. A x-ray pair distribution function study demonstrated that the structural distortion within Third Unit Cell range was found to immediately disappear when the temperature is increased across the Verwey transition, while the local structural distortion within First Unit Cell range appears to exist to high temperature of about 800 K (*13*). However, it is still challenging for us to accurately interpret the nature of the structural distortions and the short-range trimeron order.

The discovery of the electronic nematicity at the temperatures above the Verwey transition is the center of the manuscript. While the precursors of trimerons may coexist with nematic reflection over a range of temperatures, the evolution from nematicity to trimerons involves a more fundamental change in the underlying electronic structure of the material. As shown in Fig. S12, two phase-transition scenarios upon cooling are compared. Whether electronic nematic phase exists at the temperatures above the Verwey transition would result in distinctive experimental observations across the Verwey transition with fundamentally different driving mechanisms from theoretical perspective. For example, electronic nematicity (rhombohedral; cubic symmetry is already broken) can help stabilize trimeron order (monoclinic) by providing a preferred directionality for the electronic degrees of freedom that participate in trimeron formation.

On the experimental side, if we perform thermal cycling in the vicinity of the Verwey transition, that preferred direction locally in the monoclinic phase is randomly selected every time when the system is cooled down below the Verwey temperature without long-range nematicity, while the preferred direction is pre-selected by the nematic order locally through each thermal cycling. Of course, in a realistic experiment, other effects such as strain and defects could also help to pre-select the preferred direction in the monoclinic phase. Nevertheless, for those two scenarios in Fig. S12, the Verwey transition involves very different thermal fluctuations and thus they must belong to two distinct families of phase transitions, as will be shown below from the theory side.

From the theoretical point of view, the two scenarios in Fig. S12 belong to two different universality classes and they are described by different field theories. This can be demonstrated by an analogy. We can think of the trimeron ordered insulating state as a solid (crystal or smectic) formed by electrons, while the high-temperature cubic state is an electron liquid (*34*). In the top scenario in Fig. S12, the Verwey transition corresponds to a direct solid-liquid transition. In contrast, in the bottom scenario in Fig. S12, the Verwey transition corresponds to a transition between a solid/smectic and nematic fluid. From the study of classical liquid crystals, the bottom scenario is known to be highly unconventional, where nematic order parameter serves the role of a gauge field (*37*, *63*, *64*).

In summary, our finding of the electronic nematicity reveals that the Verwey transition belongs to a new universality class, different from the conventional picture. The substantial impact of the electronic nematic phase can be demonstrated experimentally and theoretically for understanding the driving mechanisms of the Verwey transition.

Charge order arrangement in cubic phase and monoclinic phase

In the cubic phase, there are four atomic layers of octahedral Fe ions in one-unit cell along the ***c*** axis in the charge ordering models. Below T_v, the unit cell is expanded into $\sqrt{2a} \times \sqrt{2a} \times 2a$ in the monoclinic phase with trimeron order. Thus, there are eight atomic layers of octahedral Fe ions along the ***c*** axis in the monoclinic phase. To simplify the charge distribution sketch, the valence states of Fe ions are rounded to the nearest integer in the low-temperature phase, as shown in (*2*). The charge distribution in each layer in the electronic nematicity phase and trimerons phase is shown in Figs. S13 and S14, respectively.

Intensity variation Bragg peaks during photoexcitation (@30K, pump fluence 4 mJ·cm^{-2})

The MeV UED experiment was taken at temperature ~ 30 K, where the structure is monoclinic with the trimeron charge ordering. We prepared a UED single-crystal sample at the [110] zone axis for intensity measurements from more Bragg / SL peaks than the [001] zone shown in Fig. 1 and Fig. 2. During the UED experiment, in order to enhance the SLs intensity, the sample was slightly tilted off the zone axis on purpose as shown in Fig. S15. All the SL peak intensities drop quickly in the first 700 fs while no obvious change can be measured from Bragg peak intensities. A slow dynamic process follows, which starts from ~ 700 fs and lasts at least a few ps in our observations, which is consistent with the timescale observed in the ultrafast x-ray diffraction study (*47*).

Based on the signal noise ratio of intensity change on each Bragg peak, we found that the intensity variations can be classified as two typical types: one type is intensity decreasing (marked in blue color); another type is intensity increasing (marked in yellow color),which are labeled in Fig. S15. The measured peaks are numbered. The equivalent (hkl) and $(\bar{h}\bar{k}\bar{l})$ reflections are labeled using the same number.

The temporal evolutions of the Bragg peaks are shown below in Fig. S16. These Bragg peaks are selected considering the signal noise ratio.

Dynamic electron diffraction simulation for UED results

To understand the peak intensity variations, we performed dynamic electron diffraction analysis considering atomic charge distribution, atomic displacements, and existence of twin variants. Since the UED sample was cut from a bulk material and thinned down to less than 100 nm using Focused Ion Beam, the thickness of the sample is larger than that of the typical cleaved 2D materials and the bulk sample could be relatively flat. That is, the precession effect is reduced, and the multiple scattering effect is enhanced. Therefore, we performed dynamic electron diffraction simulation using Bloch wave method. Due to the multiple twin variants captured in the

UED experiment, we considered the Bragg peaks coming from each twin structure. Since the diameter of electron probe is around 100 μm, we assume the fraction of domains is fixed and evenly distributed in the electron diffraction calculation. All the peaks considered in the simulation are listed in Table S4 and Table S5. Each reflection in the UED pattern is numbered, e.g., B1 (Bragg peak 1), SL1 (superlattice peak 1).

A. Intensities of Bragg peaks and SLs with different valence state of Fe ions on octahedral site

The charge ordering model for the electron diffraction calculation is based on the trimeron model at low temperature. We tuned the charge discrepancy of Fe ions in the trimeron, e.g., 2.6+, 2.4+; 2.7+, 2.3+, etc., and calculated the corresponding intensities of all the measured Bragg peaks and SLs. The calculated results are shown in the plot in Fig. S17. The calculated result indicates that charge discrepancy has notable impact on the intensity of SLs, compared with the intensity variation of Bragg peaks. The diffraction calculations reveal that the intensity change of the SL peaks mainly comes from the change in electronic order parameter and the intensity variations of the Bragg peaks can be mostly attributed to atomic displacement, i.e., lattice distortions.

B. Intensities of Bragg peaks induced by phonon modes

According to the literature research, Refs(*40*, *41*, *47*) pointed that there are three dominant phonon modes in the cubic phase involved in the phase transition: X_3 (transverse optical phonon); Δ_5 (transverse acoustic phonon) and Γ_5. X_3 and Δ_5 phonon modes correspond to the primary order parameters for the structural phase transition. To investigate each specific phonon effect in the monoclinic phase, we built atomic displacement model according to each frozen phonon mode and calculated intensities of Bragg peaks and SLs shown in Figs. S18-S20. Since these phonon modes with X_3 and Δ_5 symmetry remarkably distort the octahedral sites, in atomic distortion models, we only focus on the Fe ions located in the octahedral sites, i.e., involved in the trimeron ordering, so the Fe ions on the tetrahedral sites are omitted.

Calculation result based on Γ_5 phonon mode Figure S18 is the intensity variation induced by the Γ_5 phonon mode, which is related to the β angle changes from 90º to 90.23º without affecting the relative atomic positions in the unit cell during the phase transition as shown in Fig. S18a. In the calculation, the β angle in the monoclinic phase is changed from 90.23º to 90º step by step. The calculation result indicates that β angle variation has little impact on the reflection intensities. Figure S18b is a representative result calculated for Bragg peak 1 and SL 1. Hence Γ_5 phonon mode is not the dominant mode during the photoexcitation process.

Calculation result based on Δ_5 phonon mode Figure S19a shows atomic displacements corresponding to Δ_5 phonon mode. For the Fe ions on the octahedral site, there are total 8 Fe-O layers in one unit cell in the monoclinic phase. The right part in Fig. S19a shows the relative amplitude of the atomic displacement in each layer. We calculated the intensities of Bragg peaks after moving the atoms off the original positions. The calculated intensity variation due to atomic displacement is in Figs. S19b and S19c. The calculated intensity for each Bragg peak based on the

original monoclinic phase is the reference for the intensity normalization. The Bragg peaks present different responses to the atomic displacements in the calculation. The intensities of the Bragg peaks shown in Fig. S19b are increased after photoexcitation in our experimental measurement, but the calculated intensities of Bragg peaks #5 and #16 decrease. Likewise, the intensities of the Bragg peaks shown in Fig. S19c decrease in the experimental observation. Calculated intensity of Bragg peak #30 increases, which is not consistent with the experiment. Therefore, we can conclude that Δ_5 phonon mode is not the major contribution to the intensity changes after photoexcitation.

Calculation result based on X_3 phonon mode The calculated intensities for the Bragg peaks based on the X_3 phonon mode are shown in Fig. S20. All the trends of intensity variations are consistent with the measurement results. Hence, we can conclude that X_3 phonon mode is highly excited through the strong electron-phonon coupling upon photoexcitation.

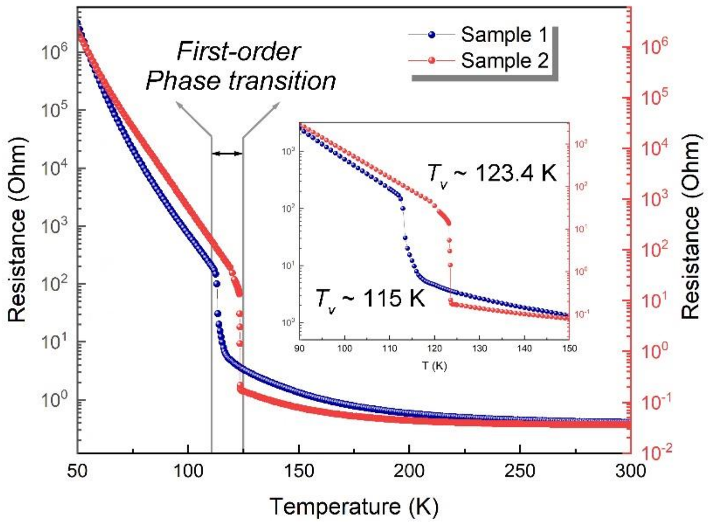

Fig. S1.

Four probe resistance measurements for Sample 1(commercial sample, SurfaceNet GmbH, Germany) and Sample 2 (from Komarek group). The samples were measured at zero field. The inset is an enlarge view of data in the vicinity of phase transition. T_v in Sample 1 is ~ 115 K, and T_v in Sample 2 is ~ 123.4 K. The measurement results demonstrate that the phase transitions in both samples are first-order phase transition, i.e., Verwey transition.

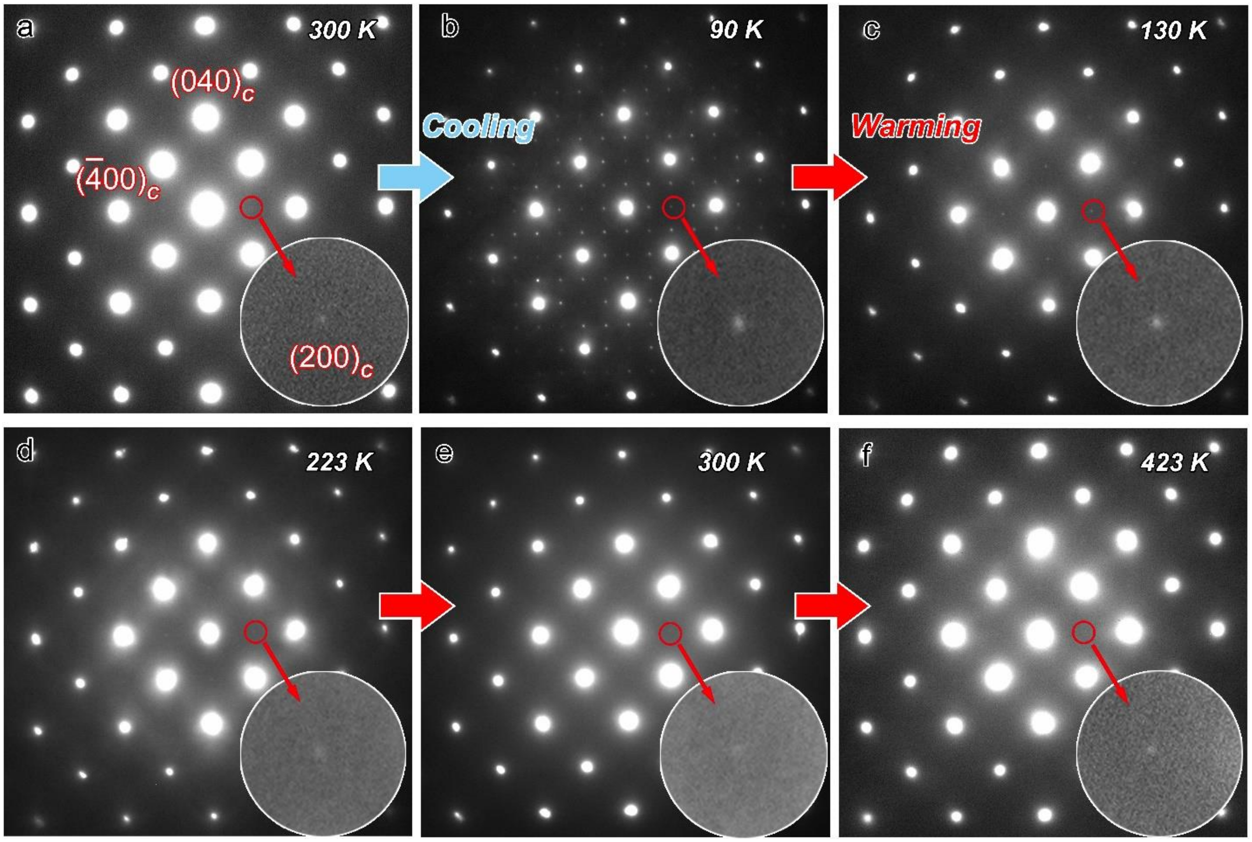

Fig. S2.

A series of electron diffraction patterns of Sample 1 along <001> zone axis taken @ 90 K, 130 K, 223 K, 300 K and 423 K. a Electron diffraction pattern taken at room temperature before cooling down, from the inverse spinel cubic phase; **b** Electron diffraction pattern from the monoclinic phase; **c-f** Diffraction patterns captured during the warming process. The inserts are the enlarged (200) peak, which is survived above the phase transition temperature.

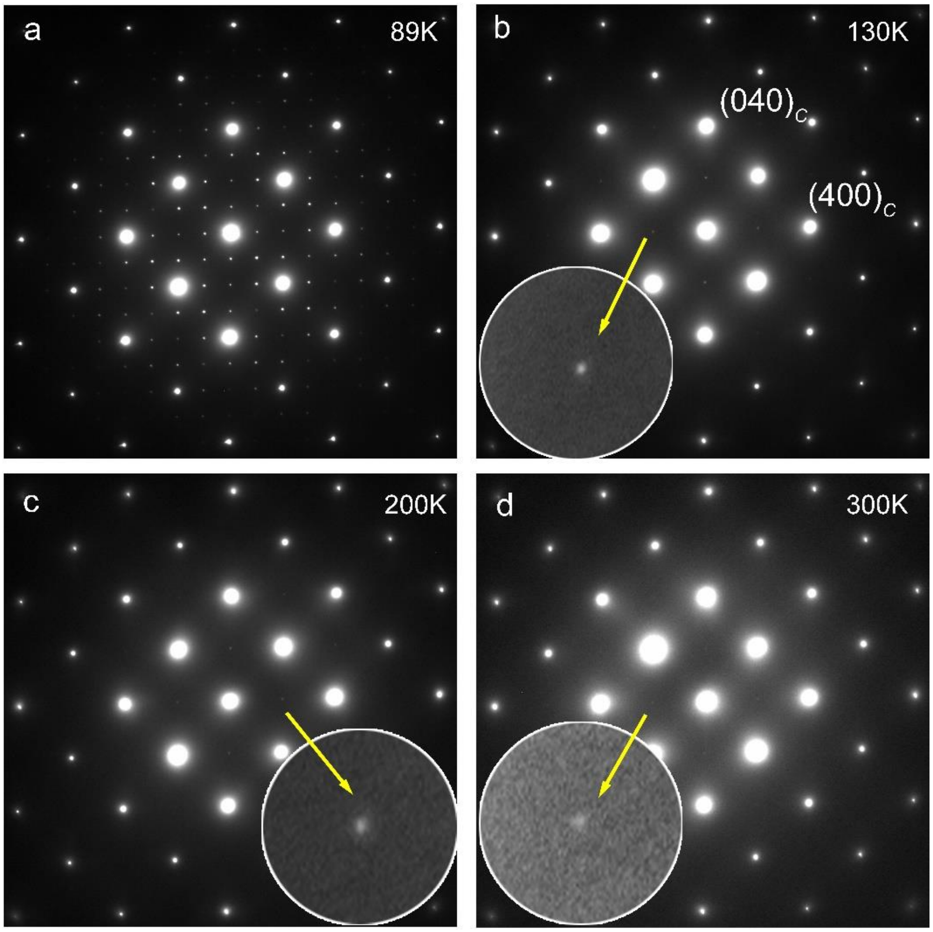

Fig. S3.

***In situ* cooling TEM experiment for Sample 2. a-d** Electron diffraction patterns along <001> zone axis during the warming process. The inserts are the persistent {200} peaks above the phase transition.

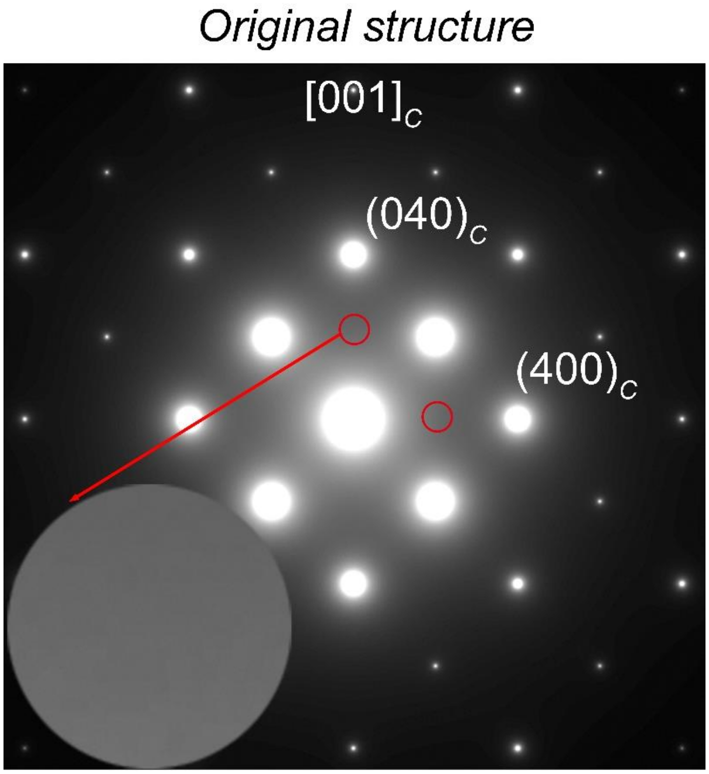

Fig. S4.
Dynamic electron diffraction simulation including the appropriate sample thickness and multiple scattering effect. Electron diffraction calculation from the standard cubic spinel structure in Fe_3O_4 along [001] direction. {200} reflections are still forbidden.

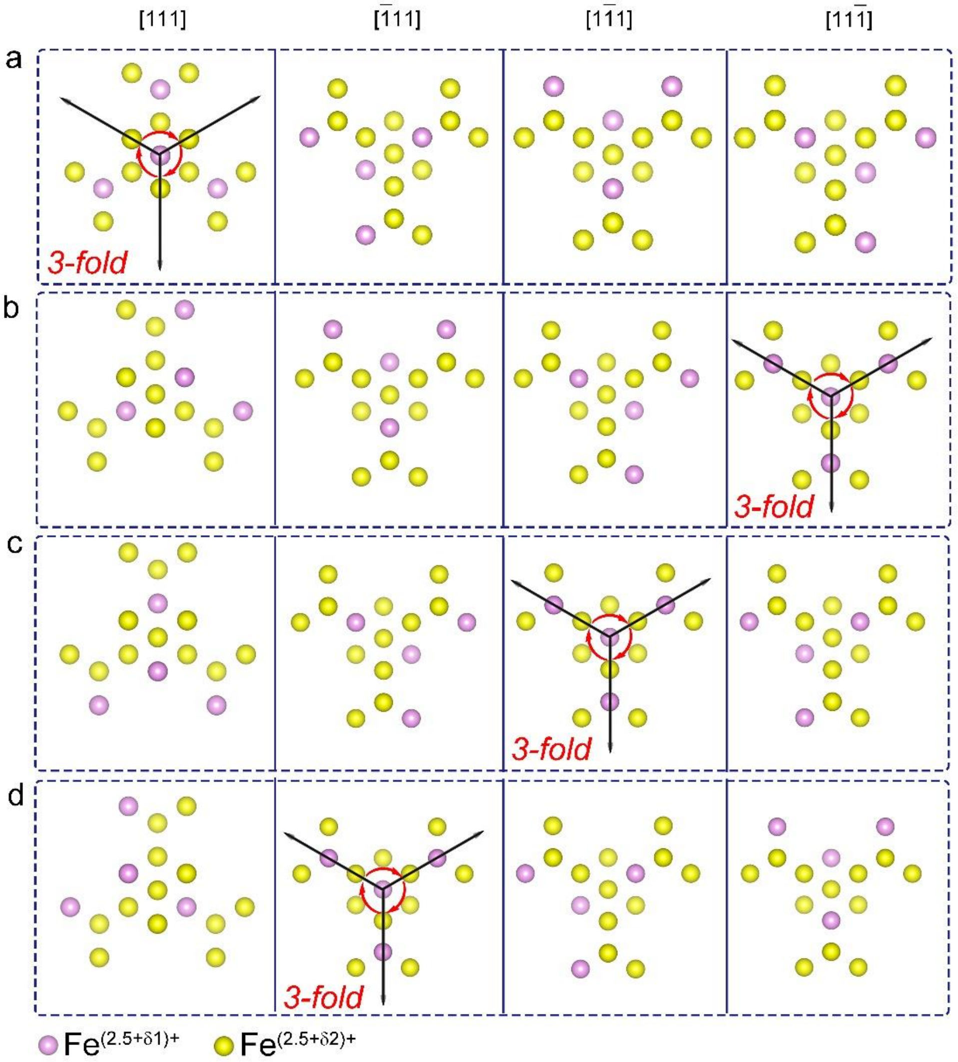

Fig. S5.

Charge distribution along <111> directions in four variants. a The valence state with $Fe^{(2.5+\delta1)+}$ is on subgroup 1 (1/2, 1/2, 1/2), the valence state of Fe ions on subgroup 2 (1/4, 3/4, 0), subgroup 3 (3/4, 0, 1/4), and subgroup 4 (0, 1/4, 3/4) is $Fe^{(2.5+\delta2)+}$. **b-d** The valence state with $Fe^{(2.5+\delta1)+}$ is on (1/4, 3/4, 0), (3/4, 0, 1/4), (0, 1/4, 3/4) subgroups, respectively. The valence state with $Fe^{(2.5+\delta2)+}$ is on the other three subgroups. In each charge ordering model, only one three-fold rotational symmetry is preserved along <111> directions, which is highlighted. Only Fe ions on octahedral sites are shown in the models.

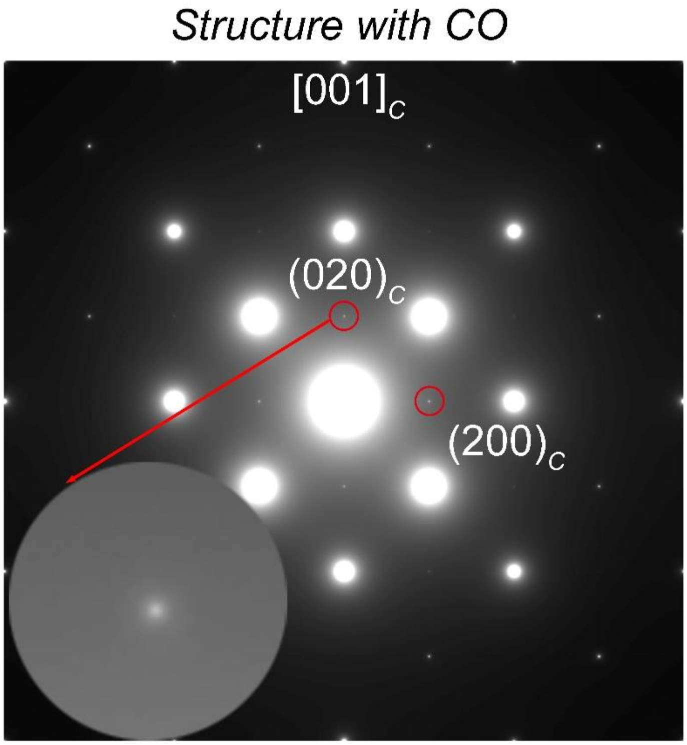

Fig. S6.
Electron diffraction simulation result. Dynamic calculation result based on the charge ordering model, (200) and (020) reflections are marked by red circles.

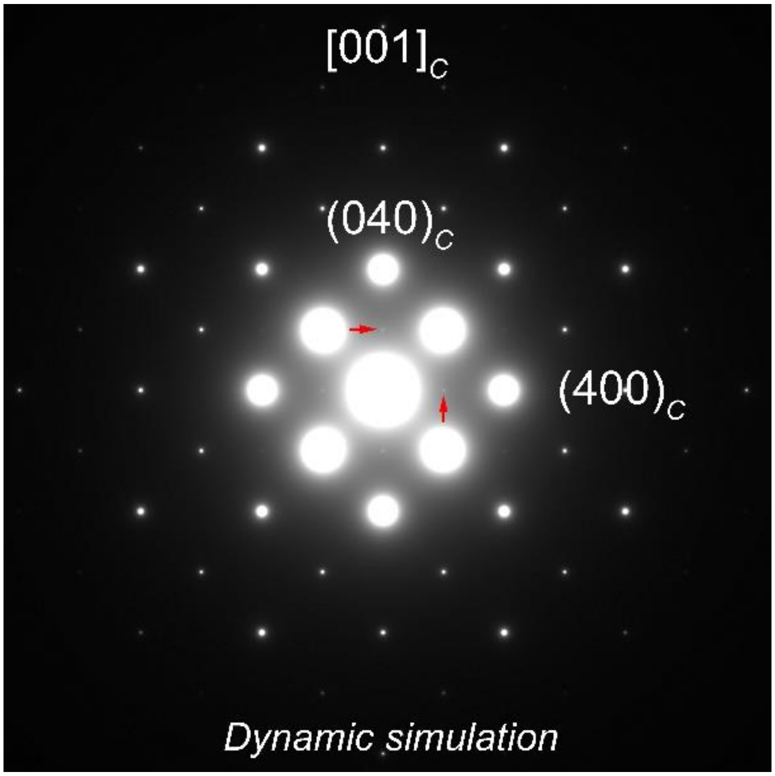

Fig. S7.

Electron diffraction simulation of atomic displacement model. The displacement is 0.005$\boldsymbol{a_c}$ and the sample thickness is 60 nm for the dynamic simulation.

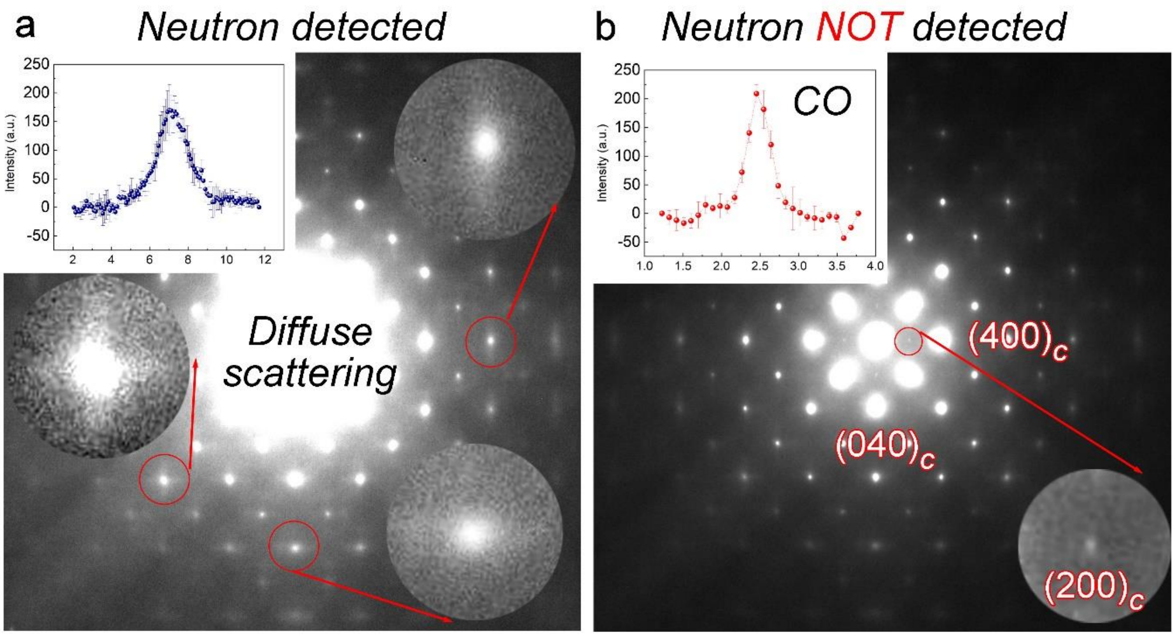

Fig. S8.
Electron diffraction pattern taken slightly above T_v. a and **b** patterns are the same diffraction pattern by changing image contrast. The diffuse scattering signal is observable in **a**. Diffuse scattering signals with different shapes are enlarged in the inserts. The insert plot is the averaged line profile intensity from these diffuse scattering signals. **b** shows the charge ordering {200} reflections. The averaged line profile intensity of these four peaks is inserted. The diffraction patterns were taken from Sample 1.

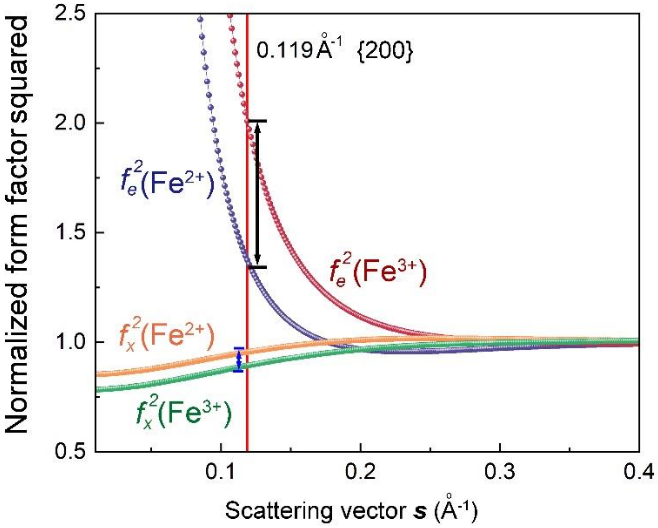

Fig. S9.

Calculated electron form factor f_e and x-ray form factor f_x for Fe^{3+} and Fe^{2+}. The normalized form factor squared (normalized by the form factor of neutral Fe atom (Fe^0) are plotted as a function of scattering vector, ***s*** (Å^{-1}). The vertical red line indicates the scattering vector of {200}. The difference of form factor squared between $f_e^2(Fe^{2+})$ and $f_e^2(Fe^{3+})$ is marked by the black arrows, and that of $f_x^2(Fe^{2+})$ and $f_x^2(Fe^{3+})$ by the blue arrows. This difference illustrates the high sensitivity of electrons to valence charge at small scattering vectors.

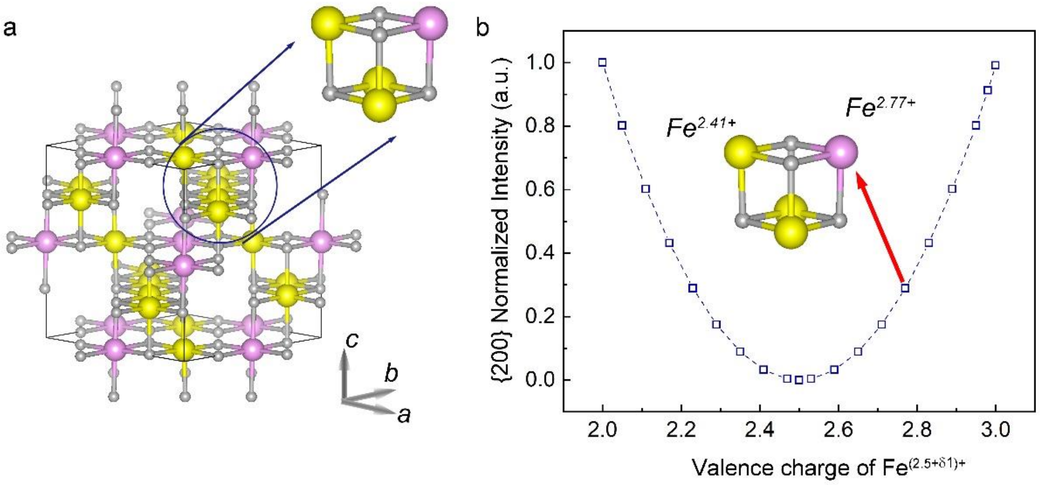

Fig. S10.

CO peak intensity calculation as a function of valence charge in cubic phase. a Crystal structure in the cubic phase, pink and yellow spheres are the Fe ions on the octahedral sites with different valence states, the grey sphere is the oxygen. The Fe ions on the tetrahedral sites are omitted. **b** Calculation result: CO {200} peak intensity as a function of the valence charge $Fe^{(2.5+\delta 1)+}$ on Fe ions.

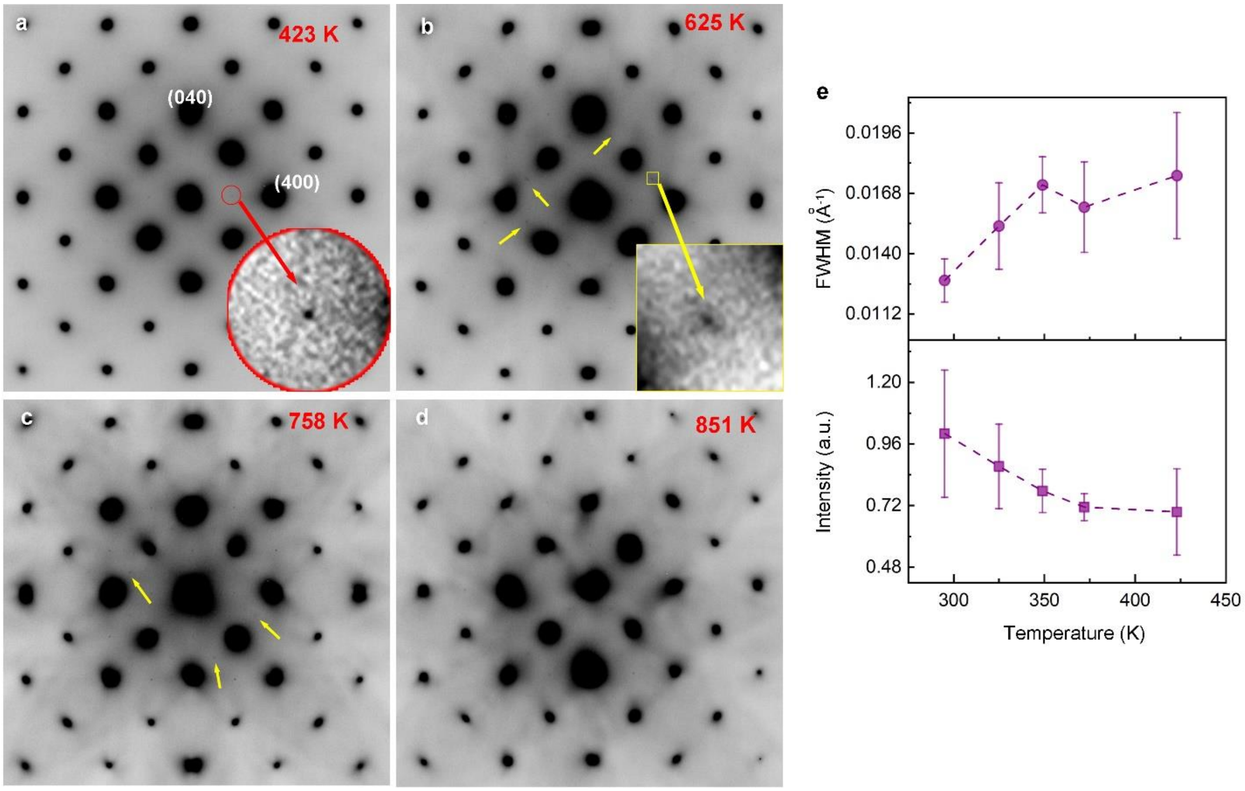

Fig. S11.

Electron diffraction patterns in the *in situ* TEM heating experiment at a 423 K, b 625 K, c 758 K and d 851 K. (200) reflection is shown in the insert in **a**. The insert in **b** is one of the extra peaks appearing at high temperatures. These extra peaks should not be observed in the cubic structure and appear upon warming. The arrows indicate the positions of the extra peak, which locates in the middle of two Bragg peaks. The arrows in **c** are used to highlight the extra peaks at 758 K. The diffuse scattering signal is strong in **c** and **d**. **e** Peak width and peak intensity of CO reflections as a function of temperature upon heating from 300 K, measured from Sample 1.

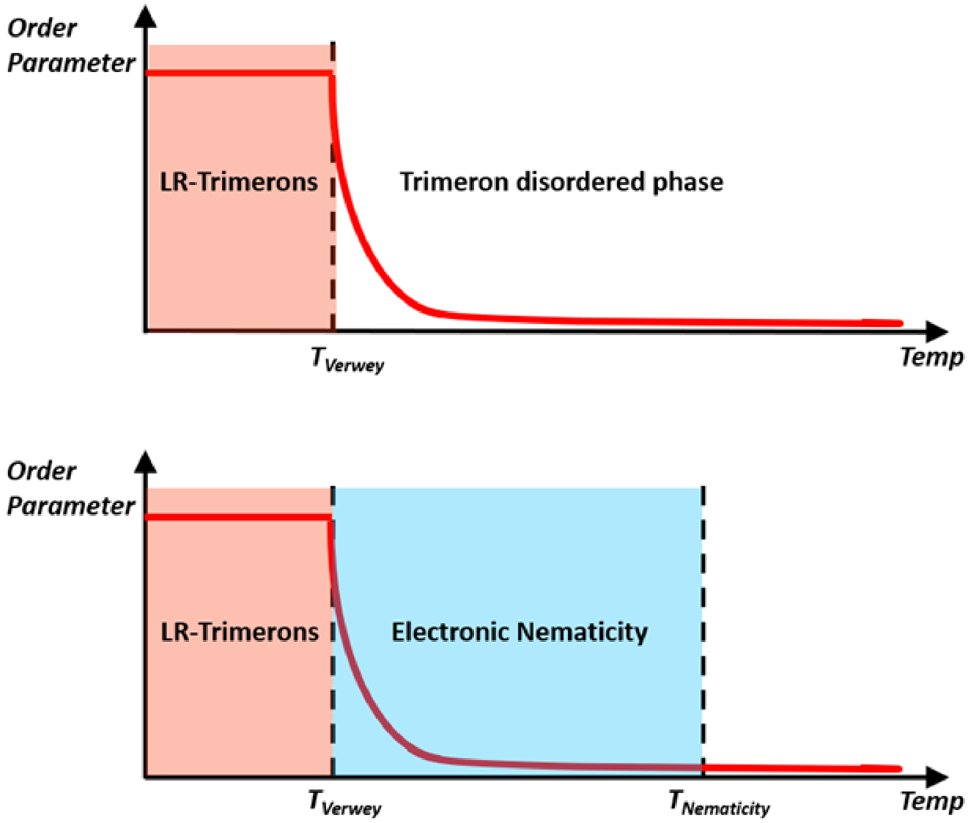

Fig. S12.

A comparison of two possible phase-transition scenarios in Fe_3O_4 upon cooling. Short-range trimerons may exist in a long-range trimeron-disordered phase with enhanced order parameter from short-range trimerons as temperature decreased (Top). Or short-range trimerons may exist in a long-range electronic nematic phase and both phases evolve with enhanced order parameters upon cooling (Bottom). Note that the plots of the order parameters (red lines) are only eye guides and may not reflect the real case quantitatively. These two scenarios would result in distinctive experimental observations across the Verwey transition with fundamentally different driving mechanisms from theoretical perspective.

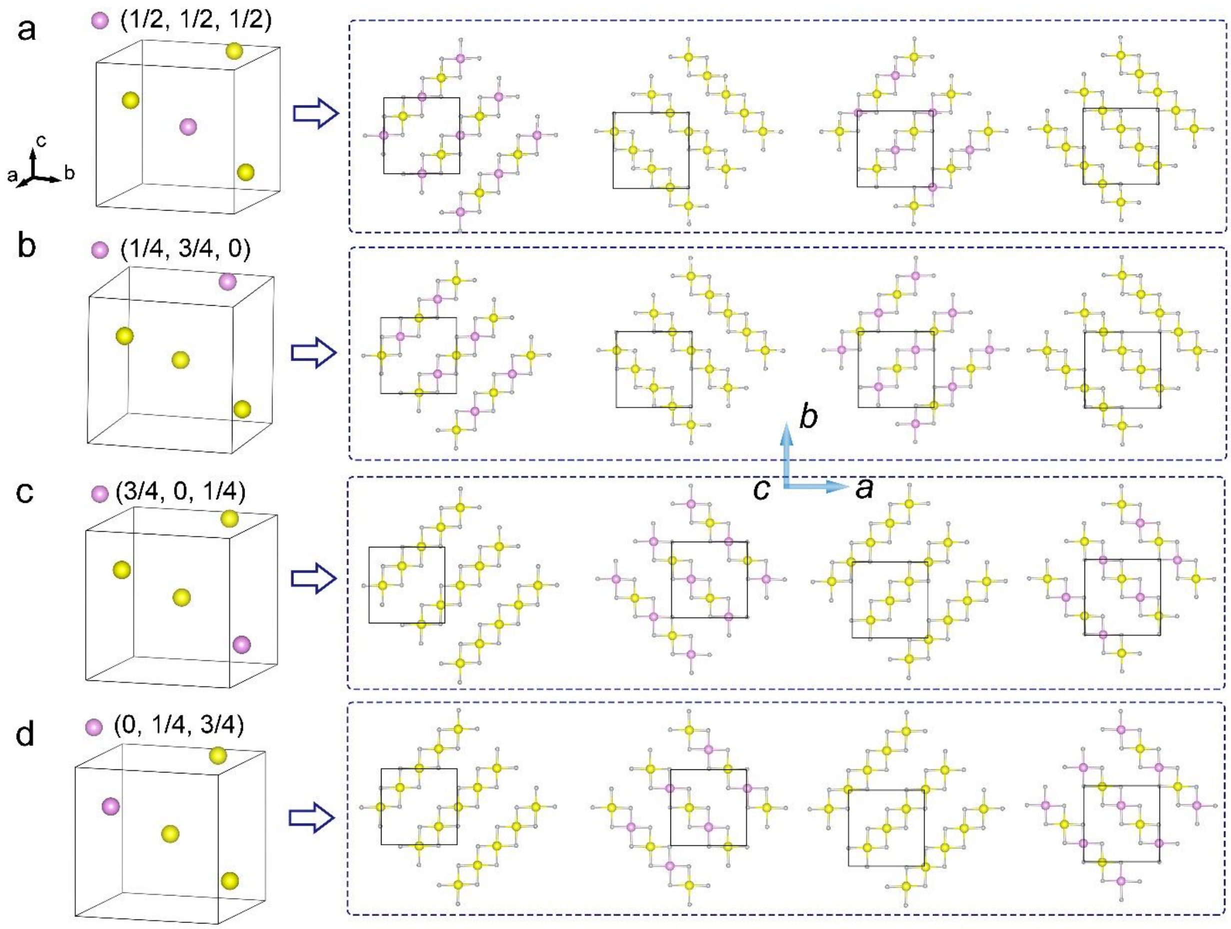

Fig. S13.

Charge order arrangement in each layer in cubic phase. a-d are four types of charge distribution. The left panel is one unit cell of cubic structure. The four Fe atoms are from octahedral sites. Other Fe atoms and oxygen atoms are omitted. The coordinates are (1/2, 1/2, 1/2), (1/4, 3/4, 0), (3/4, 0, 1/4), (0, 1/4, 3/4), which represent the four subgroups, mentioned in Table S2. In the charge ordering model, the valence charge in one subgroup is different with other three subgroups. **a** shows the valence charge of Fe atom at (1/2, 1/2, 1/2) is different with other three atoms, which is highlighted by pink color, and the valence charge is $Fe^{(2.5+\delta1)+}$. The valence charge in the other three atoms is the same, i.e., $Fe^{(2.5+\delta2)+}$, which is in yellow color. According to the face center symmetry in the cubic structure, each atom has another three equivalent atoms as shown in Table S2. There are total 16 Fe atoms in the octahedral sites, distributed in four layers in one unit cell. The unit cell is outlined by the black frame. The charge distribution in each layer along ***c*** axis is shown in the right panel. In **b**, **c** and **d**, the atom with the valence charge of $Fe^{(2.5+\delta1)+}$ locates in (1/4, 3/4, 0), (3/4, 0, 1/4), (0, 1/4, 3/4), respectively.

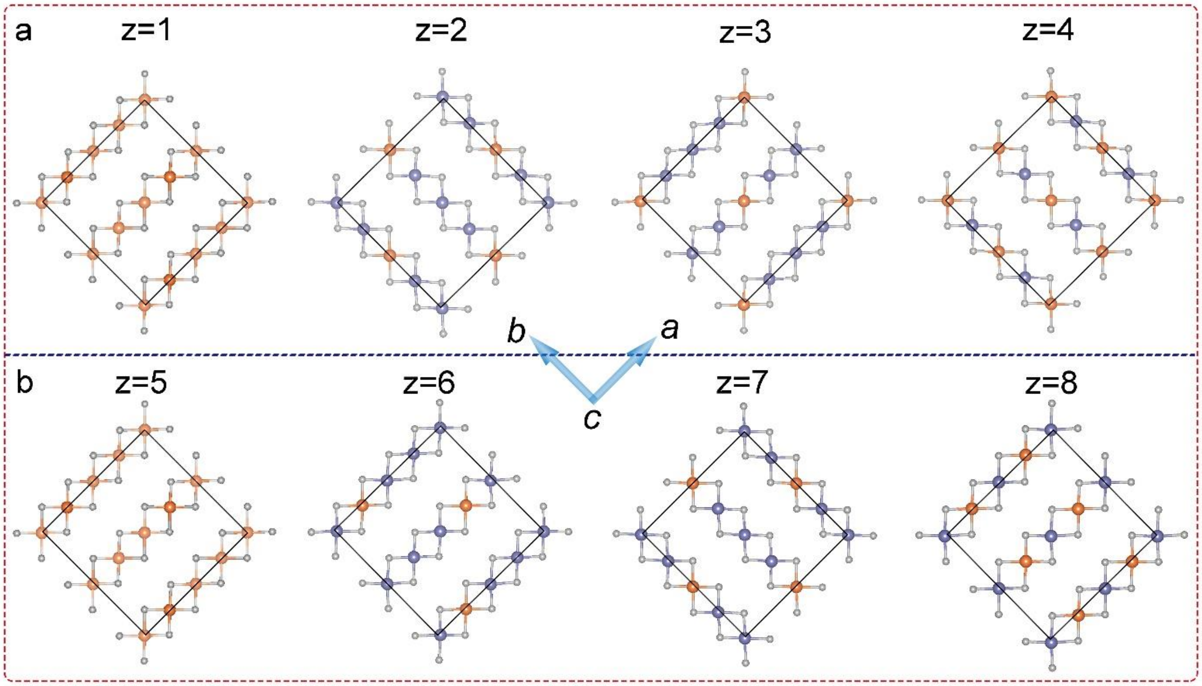

Fig. S14.
Charge order arrangement in each layer in the monoclinic phase. The measurement result is from in(*2*). In the monoclinic structure, there are eight layers of octahedral Fe ions in one-unit cell. z is the layer number along ***c*** axis. The purple and orange spheres represent Fe ions with different valence states, e.g., Fe^{3+} and Fe^{2+}, which are rounded to the nearest integer.

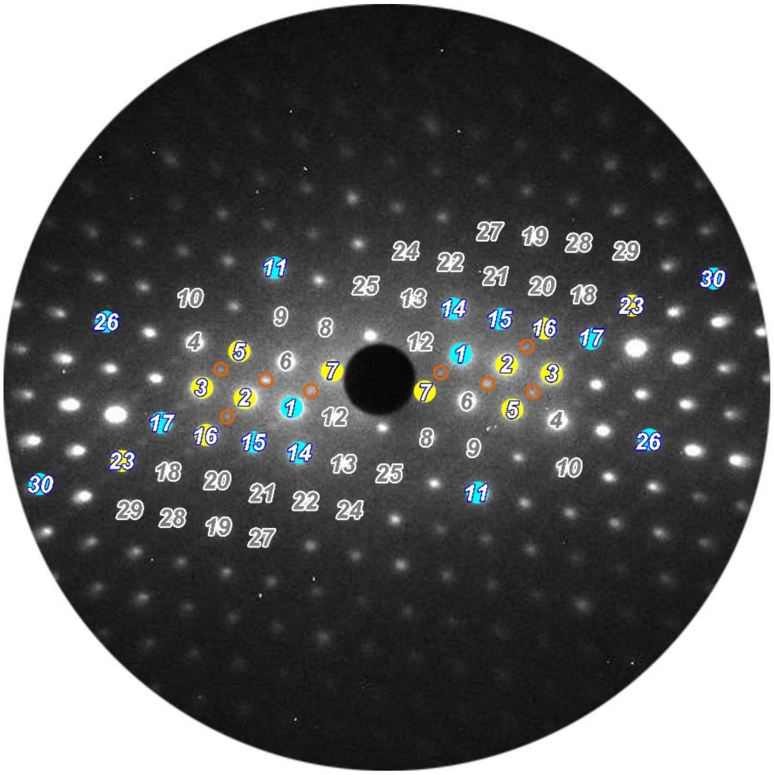

Fig. S15.

UED pattern at 30 K. The measured Bragg peaks are marked with numbers. The equivalent peaks are using the same number. The Bragg peaks shown with blue and yellow color present clear intensity decreasing and increasing tendency after photoexcitation, respectively. The SLs highlighted by circles.

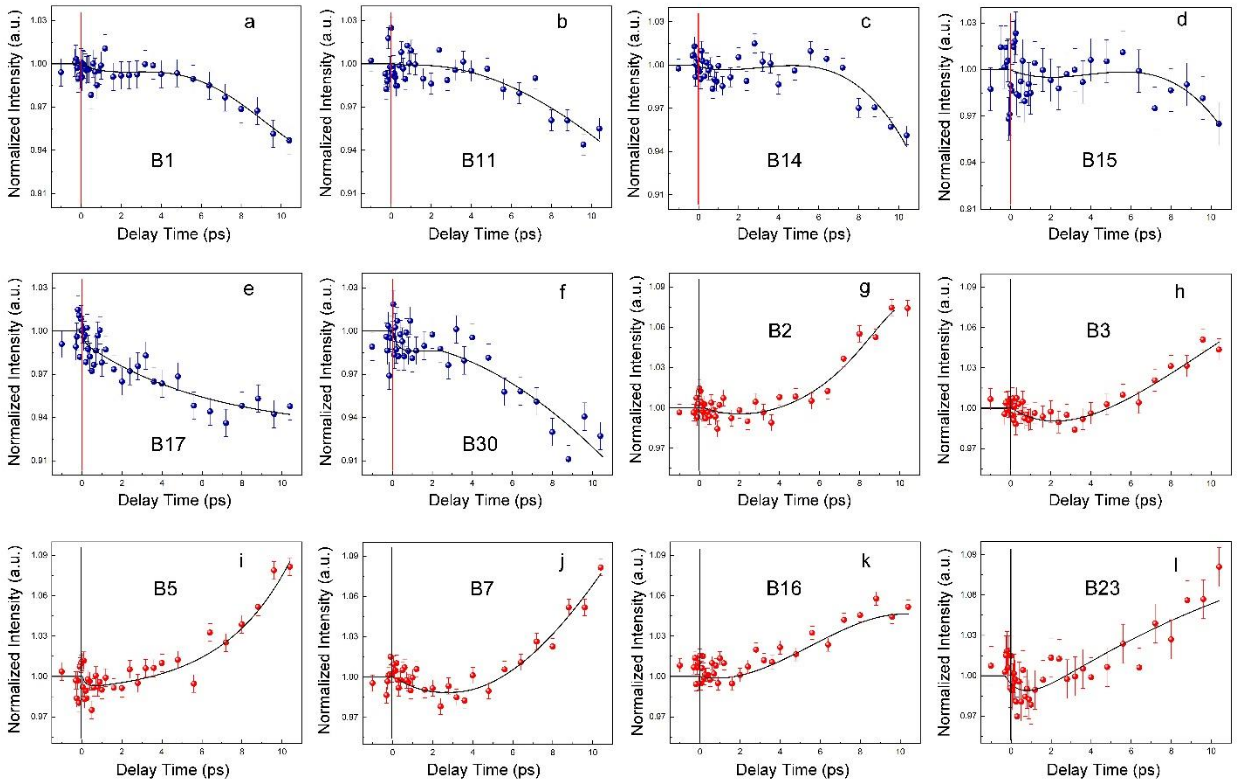

Fig. S16.

Temporal evolutions of the Bragg peaks. a-f Intensities of Bragg peaks are decreasing; **g-l** Intensities of Bragg peaks are increasing as a function of time. Error bars represent the standard deviation in the mean of intensity before time zero. The solid line is a guide to the eye.

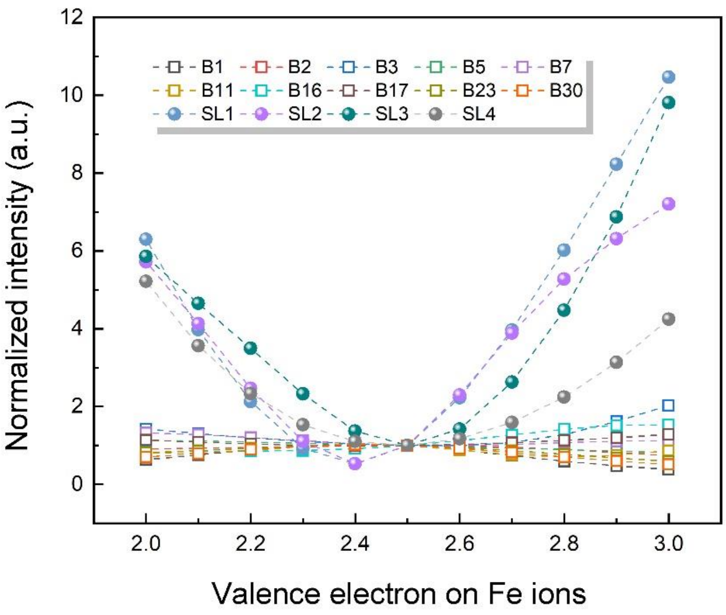

Fig. S17.

Electron diffraction calculation result on charge discrepancy on Fe ions in the monoclinic phase. The peak intensity is set to 1 as a reference, when the valence electron on the Fe ions is 2.5+, i.e., charge discrepancy is zero. The intensity variations induced by the charge discrepancy on each Bragg peak and SL are calculated as a function of charge disproportion on Fe ions.

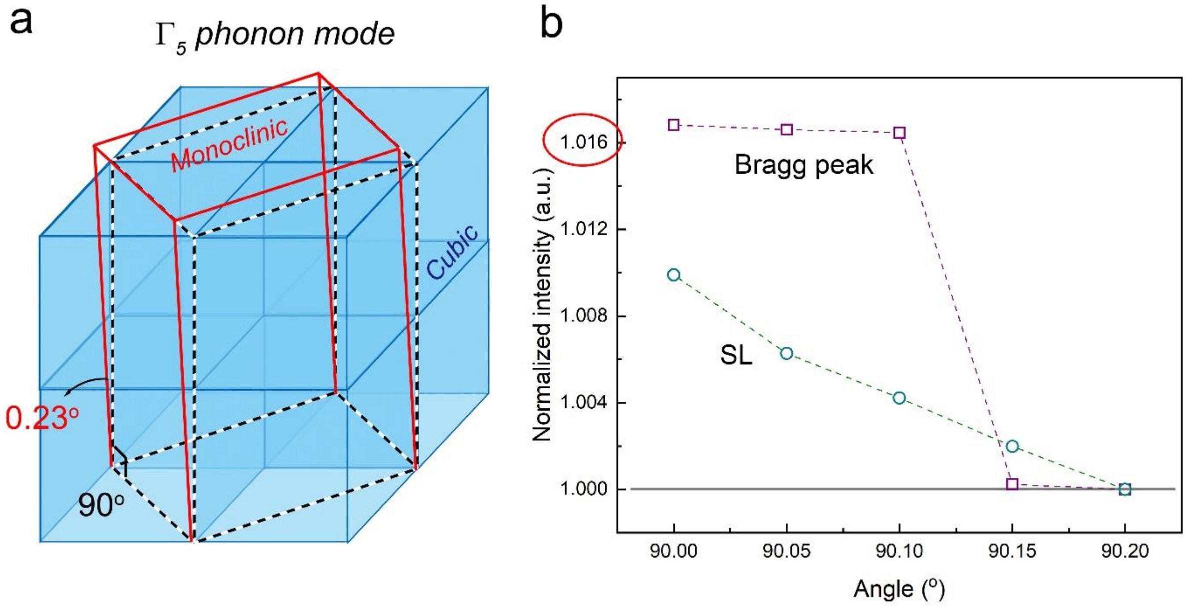

Fig. S18.

Γ_5 phonon mode. a Sketch of phonon Γ_5 mode, which is related to the β angle change during the phase transition from 90˚ in the cubic phase to 90.23˚ in the monoclinic phase. **b** Bragg peak 1 and SL 1 Intensity variation induced by angle β changing. The intensity in the monoclinic phase is the reference as 1.

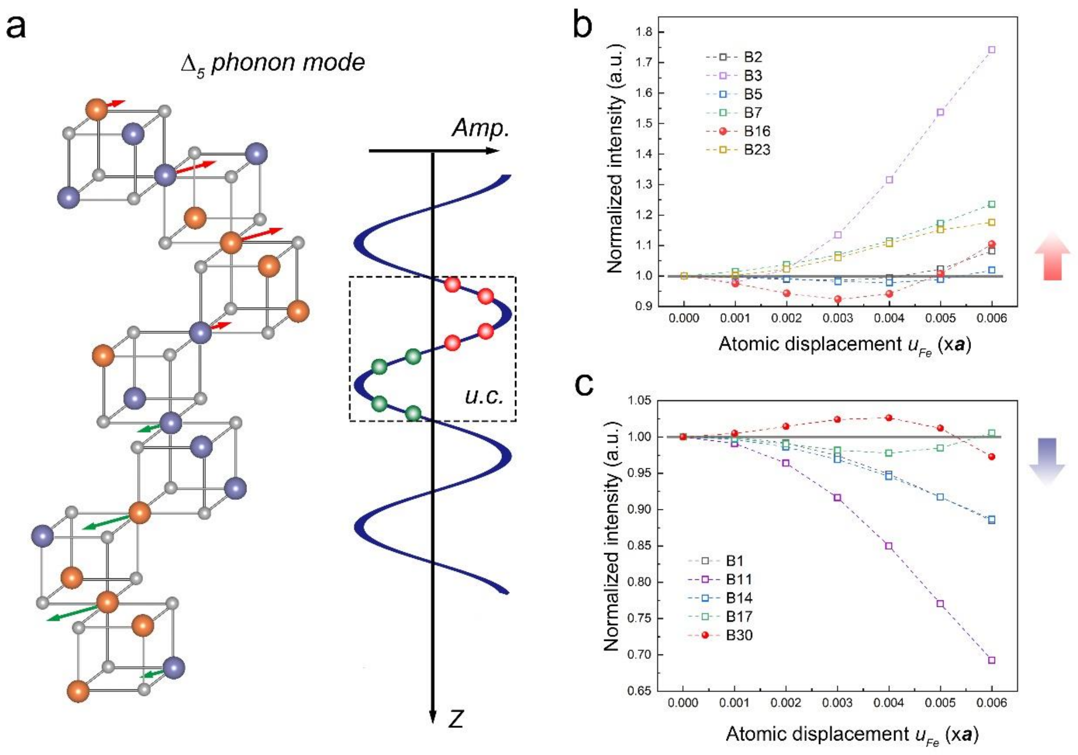

Fig. S19.

Δ_5 phonon mode. a Atomic displacements model corresponding to the Δ_5 phonon mode. The arrows present the direction of atomic displacements, and the length of arrow is proportional to the displacement amplitude, which is shown in the sine wave curve in the right part. The red dots and green dots show the relative displacement in each layer in one unit cell along ***z*** direction. **b** and **c** Calculated intensities for the Bragg peaks we studied. The red arrow indicates the measured intensity of the Bragg peaks shown in **b** are increasing as a function of time in the experiment; the bule arrow indicate the intensities of those reflection in **c** are decreasing with time in the experimental observation.

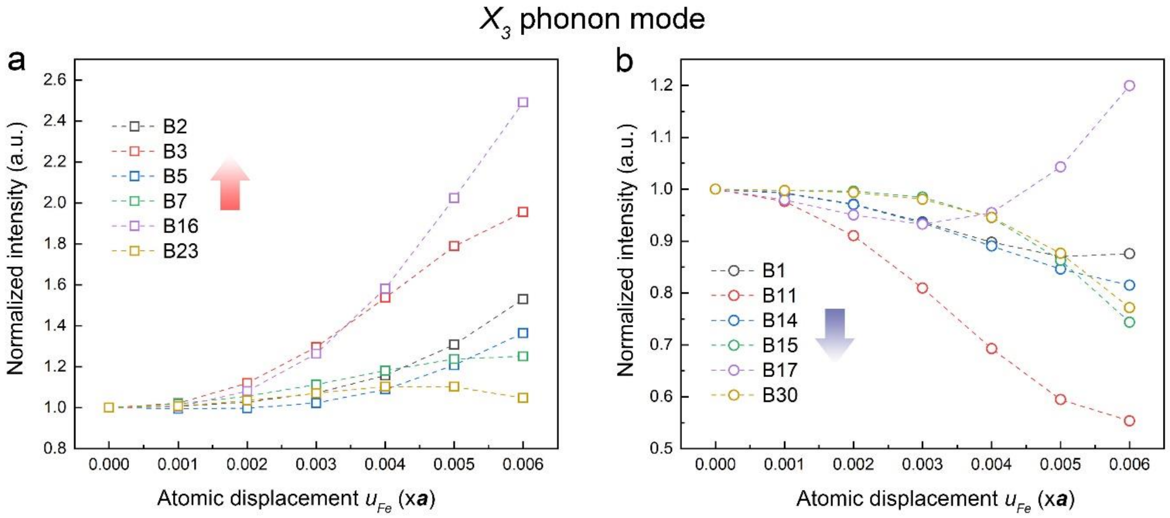

Fig. S20.

Calculation results based on X_3 phonon mode. a and **b** Calculated intensities for the Bragg peaks based on X_3 phonon mode. The red arrow indicates the measured intensity of the Bragg peaks shown in **a** are increasing as a function of time; the blue arrow indicates the intensities of those reflection in **b** are decreasing with time measured from the experimental data.

Table S1. Wyckoff position in $Fd\bar{3}m$ cubic phase

Atom	Multiplicity	x	y	z
Fe (*tet.*)	8*a*	1/8	1/8	1/8
Fe (*oct.*)	16*d*	1/2	1/2	1/2
O	32*e*	0.2549	0.2549	0.2549

Table S2. Fe Atom position in the octahedral sites

**F.C.C**		**(0, 0, 0)**	**(0, 1/2, 1/2)**	**(1/2, 0, 1/2)**	**(1/2, 1/2, 0)**
subgroup 1	f_1	(1/2, 1/2, 1/2)	(1/2, 0, 0)	(0, 1/2, 0)	(0, 0, 1/2)
subgroup 2	f_2	(1/4, 3/4, 0)	(1/4, 1/4, 1/2)	(3/4, 3/4, 1/2)	(3/4, 1/4, 0)
subgroup 3	f_3	(3/4, 0, 1/4)	(3/4, 1/2, 3/4)	(1/4, 0, 3/4)	(1/4, 1/2, 1/4)
subgroup 4	f_4	(0, 1/4, 3/4)	(0, 3/4, 1/4)	(1/2, 1/4, 1/4)	(1/2, 3/4, 3/4)

Where f_i, i =1, 2, 3, 4, is the atom form factor of Fe ions on the 16*d* site.

Table S3. Atomic displacements (δ_i):

**F.C.C**	**(0, 0, 0)**	**(0, 1/2, 1/2)**	**(1/2, 0, 1/2)**	**(1/2, 1/2, 0)**
subgroup 1	(1/2+δ_1, 1/2, 1/2)	(1/2+δ_1, 0, 0)	(δ_1, 1/2, 0)	(δ_1, 0, 1/2)
subgroup 2	(1/4+δ_2, 3/4, 0)	(1/4+δ_2, 1/4, 1/2)	(3/4+δ_2, 3/4, 1/2)	(3/4+δ_2, 1/4, 0)
subgroup 3	(3/4+δ_2, 0, 1/4)	(3/4+δ_2, 1/2, 3/4)	(1/4+δ_2, 0, 3/4)	(1/4+δ_2, 1/2, 1/4)
subgroup 4	(δ_2, 1/4, 3/4)	(δ_2, 3/4, 1/4)	(1/2+δ_2, 1/4, 1/4)	(1/2+δ_2, 3/4, 3/4)

Table S4. Bragg peaks included in the diffraction simulation

	**B1**	**B2**	**B3**	**B5**	**B7**	**B11**
$[100]_{LT}$	$(0\ 2\ \bar{6})$	$(0\ 4\ \bar{8})$	$(0\ 6\ \overline{10})$	$(0\ 6\ \bar{6})$	$(0\ 2\ \bar{2})$	$(0\ 8\ 0)$
	$(0\ \bar{2}\ 6)$	$(0\ \bar{4}\ 8)$	$(0\ \bar{6}\ 10)$	$(0\ \bar{6}\ 6)$	$(0\ \bar{2}\ 2)$	$(0\ \bar{8}\ 0)$
$[010]_{LT}$	$(2\ 0\ 6)$	$(4\ 0\ 8)$	$(6\ 0\ 10)$	$(6\ 0\ 6)$	$(2\ 0\ 2)$	$(8\ 0\ 0)$
	$(\bar{2}\ 0\ \bar{6})$	$(\bar{4}\ 0\ \bar{8})$	$(\bar{6}\ 0\ \overline{10})$	$(\bar{6}\ 0\ \bar{6})$	$(\bar{2}\ 0\ \bar{2})$	$(\bar{8}\ 0\ 0)$
$[111]_{LT}$	$(4\ \bar{2}\ \bar{2})$	$(6\ \bar{2}\ \bar{4})$	$(8\ \bar{2}\ \bar{6})$	$(6\ 0\ \bar{6})$	$(2\ 0\ \bar{2})$	$(\bar{4}\ \bar{4}\ 8)$
	$(\bar{4}\ 2\ 2)$	$(\bar{6}\ 2\ 4)$	$(\bar{8}\ 2\ 6)$	$(\bar{6}\ 0\ 6)$	$(\bar{2}\ 0\ 2)$	$(4\ 4\ \bar{8})$
$[\bar{1}11]_{LT}$	$(2\ 4\ \bar{2})$	$(2\ 6\ \bar{4})$	$(2\ 8\ \bar{6})$	$(0\ 6\ \bar{6})$	$(0\ 2\ \bar{2})$	$(4\ \bar{4}\ 8)$
	$(\bar{2}\ \bar{4}\ 2)$	$(2\ 6\ \bar{4})$	$(\bar{2}\ \bar{8}\ 6)$	$(0\ \bar{6}\ 6)$	$(0\ \bar{2}\ 2)$	$(\bar{4}\ 4\ \bar{8})$
$[1\bar{1}1]_{LT}$	$(2\ 4\ 2)$	$(2\ 6\ 4)$	$(2\ 8\ 6)$	$(0\ 6\ 6)$	$(0\ 2\ 2)$	$(\bar{4}\ 4\ 8)$
	$(\bar{2}\ \bar{4}\ \bar{2})$	$(\bar{2}\ \bar{6}\ \bar{4})$	$(\bar{2}\ \bar{8}\ \bar{6})$	$(0\ \bar{6}\ \bar{6})$	$(0\ \bar{2}\ \bar{2})$	$(4\ \bar{4}\ \bar{8})$
$[11\bar{1}]_{LT}$	$(2\ \bar{4}\ \bar{2})$	$(2\ \bar{6}\ \bar{4})$	$(2\ \bar{8}\ \bar{6})$	$(0\ 6\ 6)$	$(0\ 2\ 2)$	$(4\ 4\ 8)$
	$(\bar{2}\ 4\ 2)$	$(\bar{2}\ 6\ 4)$	$(\bar{2}\ 8\ 6)$	$(0\ \bar{6}\ \bar{6})$	$(0\ \bar{2}\ \bar{2})$	$(\bar{4}\ \bar{4}\ \bar{8})$
	**B14**	**B15**	**B16**	**B17**	**B23**	**B30**
$[100]_{LT}$	$(0\ 0\ 8)$	$(0\ 2\ \overline{10})$	$(0\ 4\ \overline{12})$	$(0\ 6\ \overline{14})$	$(0\ 6\ \overline{18})$	$(0\ 8\ \overline{20})$
	$(0\ 0\ \bar{8})$	$(0\ \bar{2}\ 10)$	$(0\ \bar{4}\ 12)$	$(0\ \bar{6}\ 14)$	$(0\ \bar{6}\ 18)$	$(0\ \bar{8}\ 20)$
$[010]_{LT}$	$(0\ 0\ 8)$	$(2\ 0\ 10)$	$(4\ 0\ 12)$	$(6\ 0\ 14)$	$(6\ 0\ 18)$	$(8\ 0\ 20)$
	$(0\ 0\ \bar{8})$	$(\bar{2}\ 0\ \overline{10})$	$(\bar{4}\ 0\ \overline{12})$	$(\bar{6}\ 0\ \overline{14})$	$(\bar{6}\ 0\ \overline{18})$	$(\bar{8}\ 0\ \overline{20})$
$[111]_{LT}$	$(4\ \bar{4}\ 0)$	$(\bar{6}\ 4\ 2)$	$(8\ \bar{4}\ \bar{4})$	$(10\ \bar{4}\ \bar{6})$	$(12\ \bar{6}\ \bar{6})$	$(14\ \bar{6}\ \bar{8})$
	$(\bar{4}\ 4\ 0)$	$(6\ \bar{4}\ \bar{2})$	$(\bar{8}\ 4\ 4)$	$(\overline{10}\ 4\ 6)$	$(\overline{12}\ 6\ 6)$	$(\overline{14}\ 6\ 8)$
$[\bar{1}11]_{LT}$	$(4\ 4\ 0)$	$(\bar{4}\ \bar{6}\ 2)$	$(4\ 8\ \bar{4})$	$(4\ 10\ \bar{6})$	$(6\ 12\ \bar{6})$	$(6\ 14\ \bar{8})$
	$(\bar{4}\ \bar{4}\ 0)$	$(4\ 6\ \bar{2})$	$(\bar{4}\ \bar{8}\ 4)$	$(\bar{4}\ \overline{10}\ 6)$	$(\bar{6}\ \overline{12}\ 6)$	$(\bar{6}\ \overline{14}\ 8)$
$[1\bar{1}1]_{LT}$	$(4\ 4\ 0)$	$(\bar{4}\ \bar{6}\ \bar{2})$	$(4\ 8\ 4)$	$(4\ 10\ 6)$	$(6\ 12\ 6)$	$(6\ 14\ 8)$
	$(\bar{4}\ \bar{4}\ 0)$	$(4\ 6\ 2)$	$(\bar{4}\ \bar{8}\ \bar{4})$	$(\bar{4}\ \overline{10}\ \bar{6})$	$(\bar{6}\ \overline{12}\ \bar{6})$	$(\bar{6}\ \overline{14}\ \bar{8})$
$[11\bar{1}]_{LT}$	$(4\ \bar{4}\ 0)$	$(4\ \bar{6}\ \bar{2})$	$(4\ \bar{8}\ \bar{4})$	$(4\ \overline{10}\ \bar{6})$	$(6\ \overline{12}\ \bar{6})$	$(6\ \overline{14}\ \bar{8})$
	$(\bar{4}\ 4\ 0)$	$(\bar{4}\ 6\ 2)$	$(\bar{4}\ 8\ 4)$	$(\bar{4}\ 10\ 6)$	$(\bar{6}\ 12\ 6)$	$(\bar{6}\ 14\ 8)$

Table S5. Superlattice peaks included in the diffraction simulation:

	SL1	SL2	SL3	SL4
$[100]_{\mathrm{LT}}$	$(0\ \bar{4}\ 6)$	$(0\ \bar{2}\ 4)$	$(0\ \bar{4}\ 10)$	$(0\ \bar{6}\ 12)$
	$(0\ 4\ \bar{6})$	$(0\ 2\ \bar{4})$	$(0\ 4\ \overline{10})$	$(0\ 6\ \overline{12})$
$[010]_{\mathrm{LT}}$	$(4\ 0\ 6)$	$(2\ 0\ 4)$	$(4\ 0\ 10)$	$(6\ 0\ 12)$
	$(\bar{4}\ 0\ \bar{6})$	$(\bar{2}\ 0\ \bar{4})$	$(\bar{4}\ 0\ \overline{10})$	$(\bar{6}\ 0\ \overline{12})$
$[111]_{\mathrm{LT}}$	$(5\ \bar{1}\ \bar{4})$	$(3\ \bar{1}\ \bar{2})$	$(7\ \bar{3}\ \bar{4})$	$(9\ \bar{3}\ \bar{6})$
	$(\bar{5}\ 1\ 4)$	$(\bar{3}\ 1\ 2)$	$(\bar{7}\ 3\ 4)$	$(\bar{9}\ 3\ 6)$
$[\bar{1}11]_{LT}$	$(1\ 5\ \bar{4})$	$(1\ 3\ \bar{2})$	$(3\ 7\ \bar{4})$	$(3\ 9\ \bar{6})$
	$(\bar{1}\ \bar{5}\ 4)$	$(\bar{1}\ \bar{3}\ 2)$	$(\bar{3}\ \bar{7}\ 4)$	$(\bar{3}\ \bar{9}\ 6)$
$[1\bar{1}1]_{LT}$	$(1\ 5\ 4)$	$(1\ 3\ 2)$	$(3\ 7\ 4)$	$(3\ 9\ 6)$
	$(\bar{1}\ \bar{5}\ \bar{4})$	$(\bar{1}\ \bar{3}\ \bar{2})$	$(\bar{3}\ \bar{7}\ \bar{4})$	$(\bar{3}\ \bar{9}\ \bar{6})$
$[11\bar{1}]_{LT}$	$(1\ \bar{5}\ \bar{4})$	$(1\ \bar{3}\ \bar{2})$	$(3\ \bar{7}\ \bar{4})$	$(3\ \bar{9}\ \bar{6})$
	$(\bar{1}\ 5\ 4)$	$(\bar{1}\ 3\ 2)$	$(\bar{3}\ 7\ 4)$	$(\bar{3}\ 9\ 6)$

REFERENCES AND NOTES

1. E. J. W. Verwey, Electronic conduction of magnetite (Fe_3O_4) and its transition point at low temperatures. *Nature* **144**, 327–328 (1939).

2. M. S. Senn, J. P. Wright, J. P. Attfield, Charge order and three-site distortions in the Verwey structure of magnetite. *Nature* **481**, 173–176 (2012).

3. E. J. W. Verwey, P. W. Haayman, Electronic conductivity and transition point of magnetite (“Fe_3O_4”). *Physica* **8**, 979–987 (1941).

4. M. Coey, Charge-ordering in oxides. *Nature* **430**, 155–157 (2004).

5. F. Walz, The Verwey transition - a topical review. *J. Phys. Condens. Matter* **14**, R285–R340 (2002).

6. J. García, G. Subías, The Verwey transition—A new perspective. *J. Phys. Condens. Matter* **16**, R145–R178 (2004).

7. Y. Fujii, G. Shirane, Y. Yamada, Study of the 123-K phase transition of magnetite by critical neutron scattering. *Phys. Rev. B.* **11**, 2036–2041 (1975).

8. K. Siratori, Y. Ishii, Y. Morii, S. Funahashi, S. Todo, A. Yanase, Neutron diffuse scattering study of the high temperature phase of Fe_3O_4- I, determination of atomic displacements at the *X* point in the Brillouin zone. *J. Phys. Soc. Jpn* **67**, 2818–2827 (1998).

9. H.-T. Jeng, G. Y. Guo, D. J. Huang, Charge-orbital ordering and Verwey transition in magnetite. *Phys. Rev. Lett.* **93**, 156403 (2004).

10. S. M. Shapiro, M. Iizumi, G. Shirane, Neutron scattering study of the diffuse critical scattering associated with the Verwey transition in magnetite Fe_3O_4. *Phys. Rev. B* **14**, 200–207 (1976).

11. Y. Yamada, N. Wakabayashi, R. M. Nicklow, Neutron diffuse scattering in magnetite due to molecular polarons. *Phys. Rev. B* **21**, 4642–4648 (1980).

12. A. Bosak, D. Chernyshov, M. Hoesch, P. Piekarz, M. Le Tacon, M. Krisch, A. Kozłowski, A. M. Oleś, K. Parlinski, Short-range correlations in magnetite above the Verwey temperature. *Phys. Rev. X* **4**, 011040 (2014).

13. G. Perversi, E. Pachoud, J. Cumby, J. M. Hudspeth, J. P. Wright, S. A. J. Kimber, J. Paul Attfield, Co-emergence of magnetic order and structural fluctuations in magnetite. *Nat. Commun.* **10**, 2857 (2019).

14. H. Elnaggar, R. Wang, S. Lafuerza, E. Paris, A. C. Komarek, H. Guo, Y. Tseng, D. McNally, F. Frati, M. W. Haverkort, M. Sikora, T. Schmitt, F. M. F. de Groot, Possible absence of trimeron correlations above the Verwey temperature in Fe_3O_4. *Phys. Rev. B* **101**, 085107 (2020).

15. P. W. Anderson, Ordering and Antiferromagnetism in Ferrites. *Phys. Rev.* **102**, 1008–1013 (1956).

16. R. Aragón, R. J. Rasmussen, J. P. Shepherd, J. W. Koenitzer, J. M. Honig, Effect of stoichiometry changes on electrical properties of magnetite. *J. Magn. Magn. Mater.* **54-57**, 1335–1336 (1986).

17. Z. Kąkol, J. M. Honig, Influence of deviations from ideal stoichiometry on the anisotropy parameters of magnetite $Fe_{3(1-\delta)}O_4$. *Phys. Rev. B* **40**, 9090–9097 (1989).

18. A. Chainani, T. Yokoya, T. Morimoto, T. Takahashi, S. Todo, High-resolution photoemission spectroscopy of the Verwey transition in Fe_3O_4. *Phys. Rev. B* **51**, 17976–17979 (1995).

19. D. Schrupp, M. Sing, M. Tsunekawa, H. Fujiwara, S. Kasai, A. Sekiyama, S. Suga, T. Muro, V. A. M. Brabers, R. Claessen, High-energy photoemission on Fe_3O_4: Small polaron physics and the Verwey transition. *Europhys. Lett.* **70**, 789–795 (2005).

20. T. K. Shimizu, J. Jung, H. S. Kato, Y. Kim, M. Kawai, Termination and Verwey transition of the (111) surface of magnetite studied by scanning tunneling microscopy and first-principles calculations. *Phys. Rev. B* **81**, 235429 (2010).

21. W. H. Bragg, The Structure of Magnetite and the Spinels. *Nature* **95**, 561–561 (1915).

22. M. E. Fleet, The structure of magnetite. *Acta Crystallogr. B* **37**, 917–920 (1981).

23. J.-H. Chu, H.-H. Kuo, J. G. Analytis, I. R. Fisher, Divergent nematic susceptibility in an iron arsenide superconductor. *Science* **337**, 710–712 (2012).

24. P. Malinowski, Q. Jiang, J. J. Sanchez, J. Mutch, Z. Liu, P. Went, J. Liu, P. J. Ryan, J.-W. Kim, J.-H. Chu, Suppression of superconductivity by anisotropic strain near a nematic quantum critical point. *Nat. Phys.* **16**, 1189–1193 (2020).

25. A. E. Böhmer, P. Burger, F. Hardy, T. Wolf, P. Schweiss, R. Fromknecht, M. Reinecker, W. Schranz, C. Meingast, Nematic susceptibility of hole-doped and electron-doped $BaFe_2As_2$ iron-based superconductors from shear modulus measurements. *Phys. Rev. Lett.* **112**, 047001 (2014).

26. S. Borroni, J. Teyssier, P. Piekarz, A. B. Kuzmenko, A. M. Oleś, J. Lorenzana, F. Carbone, Light scattering from the critical modes of the Verwey transition in magnetite. *Phys. Rev. B* **98**, 184301 (2018).

27. J. García, G. Subías, M. G. Proietti, H. Renevier, Y. Joly, J. L. Hodeau, J. Blasco, M. C. Sánchez, J. F. Bérar, Resonant "Forbidden" Reflections in Magnetite. *Phys. Rev. Lett.* **85**, 578–581 (2000).

28. T. Hahn, *Volume A: Space Group Symmetry* in *International Tables for Crystallography*, T. Hahn, Ed. (Springer); https://link.springer.com/referencework/10.1107/97809553602060000100).

29. Y. Zhu, L. Wu, J. Tafto, Accurate measurements of valence electron distribution and interfacial lattice displacement using quantitative electron diffraction. *Microsc. Microanal.* **9**, 442–456 (2003).

30. J. Fink, E. Schierle, E. Weschke, J. Geck, Resonant elastic soft x-ray scattering. *Rep. Prog. Phys.* **76**, 056502 (2013).

31. J. García, G. Subías, M. G. Proietti, J. Blasco, H. Renevier, J. L. Hodeau, Y. Joly, Absence of charge ordering below the Verwey transition temperature in magnetite. *Phys. Rev. B* **63**, 054110 (2001).

32. G. Subías, J. García, J. Blasco, J. Herrero-Martín, M. C. Sánchez, J. Orna, L. Morellón, Structural distortion, charge modulation and local anisotropies in magnetite below the Verwey transition using resonant X-ray scattering. *J. Synchrotron Radiat.* **19**, 159–173 (2012).

33. E. Fradkin, S. A. Kivelson, M. J. Lawler, J. P. Eisenstein, A. P. Mackenzie, Nematic fermi fluids in condensed matter physics. *Annu. Rev. Condens. Matter Phys.* **1**, 153–178 (2010).

34. S. A. Kivelson, E. Fradkin, V. J. Emery, Electronic liquid-crystal phases of a doped Mott insulator. *Nature* **393**, 550–553 (1998).

35. J. M. Tranquada, B. J. Sternlieb, J. D. Axe, Y. Nakamura, S. Uchida, Evidence for stripe correlations of spins and holes in copper oxide superconductors. *Nature* **375**, 561–563 (1995).

36. J. Wu, A. T. Bollinger, X. He, I. Božović, Spontaneous breaking of rotational symmetry in copper oxide superconductors. *Nature* **547**, 432–435 (2017).

37. P. M. Chaikin, T. C. Lubensky, *Principles of Condensed Matter Physics* (Cambridge Univ. Press, 2012; www.cambridge.org/core/books/principles-of-condensed-matter-physics/70C3D677A9B5BEC4A77CBBD0A8A23E64).

38. P. G. de Gennes, J. Prost, *The Physics of Liquid Crystals* (Clarendon Press, 1993).

39. E. Fradkin, S. A. Kivelson, J. M. Tranquada, Colloquium: Theory of intertwined orders in high temperature superconductors. *Rev. Mod. Phys.* **87**, 457–482 (2015).

40. P. Piekarz, K. Parlinski, A. M. Oleś, Mechanism of the Verwey transition in magnetite. *Phys. Rev. Lett.* **97**, 156402 (2006).

41. P. Piekarz, K. Parlinski, A. M. Oleś, Origin of the Verwey transition in magnetite: Group theory, electronic structure, and lattice dynamics study. *Phys. Rev. B* **76**, 165124 (2007).

42. K. Yamauchi, T. Fukushima, S. Picozzi, Ferroelectricity in multiferroic magnetite Fe_3O_4 driven by noncentrosymmetric Fe^{2+}/Fe^{3+} charge-ordering: First-principles study. *Phys. Rev. B* **79**, 212404 (2009).

43. E. Baldini, C. A. Belvin, M. Rodriguez-Vega, I. O. Ozel, D. Legut, A. Kozłowski, A. M. Oleś, K. Parlinski, P. Piekarz, J. Lorenzana, G. A. Fiete, N. Gedik, Discovery of the soft electronic modes of the trimeron order in magnetite. *Nat. Phys.* **16**, 541–545 (2020).

44. S. K. Park, T. Ishikawa, Y. Tokura, Charge-gap formation upon the Verwey transition in Fe_3O_4. *Phys. Rev. B* **58**, 3717–3720 (1998).

45. S. Borroni, E. Baldini, V. M. Katukuri, A. Mann, K. Parlinski, D. Legut, C. Arrell, F. van Mourik, J. Teyssier, A. Kozlowski, P. Piekarz, O. V. Yazyev, A. M. Oleś, J. Lorenzana, F. Carbone, Coherent generation of symmetry-forbidden phonons by light-induced electron-phonon interactions in magnetite. *Phys. Rev. B* **96**, 104308 (2017).

46. I. Leonov, A. N. Yaresko, V. N. Antonov, V. I. Anisimov, Electronic structure of charge-ordered Fe_3O_4 from calculated optical, magneto-optical Kerr effect, and OK-edge x-ray absorption spectra. *Phys. Rev. B* **74**, 165117 (2006).

47. S. de Jong, R. Kukreja, C. Trabant, N. Pontius, C. F. Chang, T. Kachel, M. Beye, F. Sorgenfrei, C. H. Back, B. Bräuer, W. F. Schlotter, J. J. Turner, O. Krupin, M. Doehler, D. Zhu, M. A. Hossain, A. O. Scherz, D. Fausti, F. Novelli, M. Esposito, W. S. Lee, Y. D. Chuang, D. H. Lu, R. G. Moore, M. Yi, M. Trigo, P. Kirchmann, L. Pathey, M. S. Golden, M. Buchholz, P. Metcalf, F. Parmigiani, W. Wurth, A. Föhlisch, C. Schüßler-Langeheine, H. A. Dürr, Speed limit of the insulator–metal transition in magnetite. *Nat. Mater.* **12**, 882–886 (2013).

48. F. Randi, I. Vergara, F. Novelli, M. Esposito, M. Dell'Angela, V. A. M. Brabers, P. Metcalf, R. Kukreja, H. A. Dürr, D. Fausti, M. Grüninger, F. Parmigiani, Phase separation in the nonequilibrium Verwey transition in magnetite. *Phys. Rev. B* **93**, 054305 (2016).

49. K. Sokolowski-Tinten, C. Blome, J. Blums, A. Cavalleri, C. Dietrich, A. Tarasevitch, I. Uschmann, E. Förster, M. Kammler, M. Horn-von-Hoegen, D. von der Linde, Femtosecond X-ray measurement of coherent lattice vibrations near the Lindemann stability limit. *Nature* **422**, 287–289 (2003).

50. S. Gerber, S.-L. Yang, D. Zhu, H. Soifer, J. A. Sobota, S. Rebec, J. J. Lee, T. Jia, B. Moritz, C. Jia, A. Gauthier, Y. Li, D. Leuenberger, Y. Zhang, L. Chaix, W. Li, H. Jang, J.-S. Lee, M. Yi, G. L. Dakovski, S. Song, J. M. Glownia, S. Nelson, K. W. Kim, Y.-D. Chuang, Z. Hussain, R. G. Moore, T. P. Devereaux, W.-S. Lee, P. S. Kirchmann, Z.-X. Shen, Femtosecond electron-phonon lock-in by photoemission and x-ray free-electron laser. *Science* **357**, 71–75 (2017).

51. M. Iizumi, T. F. Koetzle, G. Shirane, S. Chikazumi, M. Matsui, S. Todo, Structure of magnetite (Fe_3O_4) below the Verwey transition temperature. *Acta Crystallogr. B* **38**, 2121–2133 (1982).

52. J. P. Wright, J. P. Attfield, P. G. Radaelli, Charge ordered structure of magnetite Fe_3O_4 below the Verwey transition. *Phys. Rev. B* **66**, 214422 (2002).

53. M. Hoesch, P. Piekarz, A. Bosak, M. Le Tacon, M. Krisch, A. Kozłowski, A. M. Oleś, K. Parlinski, Anharmonicity due to electron-phonon coupling in magnetite. *Phys. Rev. Lett.* **110**, 207204 (2013).

54. H. P. Pinto, S. D. Elliott, Mechanism of the Verwey transition in magnetite: Jahn–Teller distortion and charge ordering patterns. *J. Phys. Condens. Matter* **18**, 10427–10436 (2006).

55. S. Borroni, G. S. Tucker, F. Pennacchio, J. Rajeswari, U. Stuhr, A. Pisoni, J. Lorenzana, H. M. Rønnow, F. Carbone, Mapping the lattice dynamical anomaly of the order parameters across the Verwey transition in magnetite. *New J. Phys.* **19**, 103013 (2017).

56. B. Lorenz, D. Ihle, Electron–phonon versus coulomb interaction effects at the Verwey transition of Fe_3O_4. *Phys. Status Solidi B.* **96**, 659–669 (1979).

57. J. Li, J. Li, K. Sun, L. Wu, R. Li, J. Yang, X. Shen, X. Wang, H. Luo, R. J. Cava, I. K. Robinson, X. Jin, W. Yin, Y. Zhu, J. Tao, Concurrent probing of electron-lattice dephasing induced by photoexcitation in 1T-TaSeTe using ultrafast electron diffraction. *Phys. Rev. B* **101**, 100304 (2020).

58. P. Zhu, Y. Zhu, Y. Hidaka, L. Wu, J. Cao, H. Berger, J. Geck, R. Kraus, S. Pjerov, Y. Shen, R. I. Tobey, J. P. Hill, X. J. Wang, Femtosecond time-resolved MeV electron diffraction. *New J. Phys.* **17**, 063004 (2015).

59. S. P. Weathersby, G. Brown, M. Centurion, T. F. Chase, R. Coffee, J. Corbett, J. P. Eichner, J. C. Frisch, A. R. Fry, M. Gühr, N. Hartmann, C. Hast, R. Hettel, R. K. Jobe, E. N. Jongewaard, J. R. Lewandowski, R. K. Li, A. M. Lindenberg, I. Makasyuk, J. E. May, D. McCormick, M. N. Nguyen, A. H. Reid, X. Shen, K. Sokolowski-Tinten, T. Vecchione, S. L. Vetter, J. Wu, J. Yang, H. A. Dürr, X. J. Wang, Mega-electron-volt ultrafast electron diffraction at SLAC national accelerator laboratory. *Rev. Sci. Instrum.* **86**, 073702 (2015).

60. E. J. Kirkland, *Advanced Computing in Electron Microscopy* (Springer International Publishing, Cham, 2020; http://link.springer.com/10.1007/978-3-030-33260-0).

61. J. M. Zuo, J. Pacaud, R. Hoier, J. C. H. Spence, Experimental measurement of electron diffuse scattering in magnetite using energy-filter and imaging plates. *Micron* **31**, 527–532 (2000).

62. P. G. de Gennes, An analogy between superconductors and smectics A. *Solid State Commun.* **10**, 753–756 (1972).

63. B. I. Halperin, T. C. Lubensky, S. Ma, First-order phase transitions in superconductors and Smectic-A liquid crystals. *Phys. Rev. Lett.* **32**, 292–295 (1974).
